# Potassium Intake Prevents the Induction of the Renin-Angiotensin System and Increases Medullary ACE2 and COX-2 in the Kidneys of Angiotensin II-Dependent Hypertensive Rats

**DOI:** 10.3389/fphar.2019.01212

**Published:** 2019-10-15

**Authors:** Alexis A. Gonzalez, Matias Gallardo, Carlos Cespedes, Carlos P. Vio

**Affiliations:** ^1^Institute of Chemistry, Pontificia Universidad Católica de Valparaíso, Valparaíso, Chile; ^2^Department of Physiology, Center for Aging and Regeneration CARE UC, Pontificia Universidad Católica de Chile, Santiago, Chile; ^3^Facultad de Medicina y Ciencia, Universidad San Sebastián, Santiago, Chile

**Keywords:** potassium, blood pressure, renin, angiotensin, hypertension, kallikrein, sodium, cyclooxygenase

## Abstract

In angiotensin II (Ang II)-dependent hypertensive rats there is an increased expression of proximal tubule angiotensinogen (AGT), collecting duct renin and angiotensin converting enzyme (ACE), which contributes to intratubular Ang II formation. Ang II acts on Ang II type 1 receptors promoting sodium retention and vasoconstriction. However concurrently, the ACE2-Ang-(1–7) axis and the expression of kallikrein and medullary prostaglandins counteract the effects of Ang II, promoting natriuresis and vasodilation. Human studies demonstrate that dietary potassium (K^+^) intake lowers blood pressure. In this report we evaluate the expression of AGT, ACE, medullary prorenin/renin, ACE2, kallikrein and cyclooxygenase-2 (COX-2) in Ang II-infused rats fed with high K^+^ diet (2%) for 14 days. Dietary K^+^ enhances diuresis in non-infused and in Ang II-infused rats. The rise in systolic blood pressure in Ang II-infused rats was attenuated by dietary K^+^. Ang II-infused rats showed increased renal protein levels of AGT, ACE and medullary prorenin and renin. This effect was attenuated in the Ang II + K^+^ group. Ang II infusion decreased ACE2 compared to the control group; however, K^+^ diet prevented this effect in the renal medulla. Furthermore, medullary COX-2 was dramatically induced by K^+^ diet in non-infused and in Ang II infused rats. Dietary K^+^ greatly increased kallikrein immunostaining in normotensive rats and in Ang II-hypertensive rats. These results indicate that a high K^+^ diet attenuates Ang II-dependent hypertension by preventing the induction of ACE, AGT and collecting duct renin and by enhancing medullary COX-2 and ACE2 protein expression in the kidney.

## Introduction

The activity of the systemic renin-angiotensin system (RAS) promotes vasoconstriction and sodium reabsorption primarily by angiotensin II (Ang II)-dependent stimulation of aldosterone release and by activation of distal nephron sodium channels (Masilamani et al., 1999; Chaszczewska-Markowska et al., 2016). Sodium reabsorption in the collecting duct (CD) also occurs *via* direct activation of epithelial sodium channels (ENaC) by Ang II type 1 receptor (AT1R) independent of aldosterone (Mamenko et al., 2012; Zaika et al., 2013). The amount of sodium reabsorbed by the CD impacts sodium balance and blood pressure (Kim et al., 2007) and the availability of intrarenal and intratubular Ang II is crucial for sodium entry and global sodium balance. Accumulated evidence demonstrated the presence of an intrarenal RAS (Navar et al., 2011) whose activity is stimulated in Ang II-dependent hypertension (Liu et al., 2011) and in salt induced renal injury (Kobori et al., 2001; Prieto-Carrasquero et al., 2004; Susic et al., 2009). mRNA angiotensinogen (AGT) expression can be detected in the proximal tubules and is augmented in Ang II-infused rats (Kobori et al., 2004). Renin is expressed in the CD and is upregulated in Ang II-dependent hypertension (Prieto-Carrasquero et al., 2004). This evidence, along with the augmented expression of angiotensin converting enzyme (ACE) in animal models of hypertension (Gonzalez-Villalobos et al., 2009), indicates that intrarenal RAS activation has a role in intratubular Ang II formation. Furthermore in Ang II-infused rats the concentrations of intratubular Ang II are much higher than expected by plasma accumulation (Vonthun et al., 1994; Navar et al., 2001) suggesting a critical role of the newly formed intratubular Ang II on sodium reabsorption and blood pressure.

On the other hand, the angiotensin converting enzyme 2 (ACE2) plays an important role in counteracting the effects of RAS activation (Bader, 2013). ACE2 cleaves Ang II to produce Ang-(1-7), promoting natriuresis and vasodilation *via* nitric oxide (NO) production in renal tissues through the actions of the Mas receptor (Ferrario and Varagic, 2010). Along with this axis, the Ang II infusion also promotes the induction of cyclooxygenase-2 (COX-2) in the renal medulla leading to the production of natriuretic and vasodilatory prostaglandins (PGs) counteracting the anti-natriuretic effects of Ang II.

Evidence demonstrates that K^+^ supplementation lowers blood pressure (Geleijnse et al., 2003; Rodrigues et al., 2014). This fact is relevant in light of evidence of reduced K^+^ consumption by modern society (Noubiap et al., 2015; Jung et al., 2019). Research into this matter could lead to public health interventions to prevent cardiovascular disease linked to hypertension and renal disease. Despite growing evidence showing the augmented intrarenal expression of most of the components of the RAS in Ang II-dependent hypertension (Navar et al., 2011), little is known about the effect of K^+^ diet supplementation in this model and its impact on the expression of AGT, ACE, CD renin and ACE2. Here, we investigate the effect of K^+^ dietary supplementation on systolic blood pressure, natriuresis, and protein levels of intrarenal RAS and on ACE2 and COX-2 in Ang II-dependent hypertensive rats.

## Materials and Methods

### Animals

Animal protocols were approved by the Animal Care and Use Committee of *Pontificia Universidad Católica de Chile* (Animal Welfare Assurance no. A5848-01) and conducted in accordance with the National Institutes of Health *Guide for the Care and Use of Laboratory Animals*. Six male Sprague-Dawley rats were used for each experimental group with a total number of 24 rats for this study. By the completion of the experimental protocols (14 days training on systolic blood pressure measurements, K^+^ diet, osmotic minipump implantation and 14 days of Ang II infusion), no signs of disease or mortality were observed.

### Potassium Diet

Male Sprague-Dawley rats were divided into four groups, Control group: sham-operated with normal K^+^ diet (0.9% KCl in food); Normal K^+^ diet plus chronic Ang II infusions for 14 days; High K^+^ diet with sham surgery (2% KCl in food); High K^+^ diet with chronic Ang II infusion for 14 days. Rats receiving high K^+^ diet were adapted 1 week before the beginning of treatment with 1% KCl in the drinking water.

### Ang II Infusion and Systolic Blood Pressure

Male Sprague-Dawley rats were infused subcutaneously with Ang II (Sigma, Cat # A9525, St. Louis, MO) by osmotic minipumps (Alzet model 2002, infusion rate of 0.5 mL/h) at a concentration of 400 ng/kg/min for 14 days, as previously described (Gonzalez et al., 2011). Minipump implantation was performed under ketamine/xylazyne anesthesia (25/2.5 mg/kg ip). Systolic blood pressures were monitored by the CODA tail-cuff blood pressure system (Kent Scientific Corporation, Torrington, CT) on days −7, −1, 7, and 14. At the end of the study, rats were placed during 18 h in metabolic cages (Tecniplast, Buguggiate, VA, Italy) for urine collection with the purpose of determining sodium and K^+^ concentrations and creatinine levels. After 14 days rats were euthanized by anesthetic overdose and blood samples were collected from the vena cava for electrolytes and creatinine measurements to calculate fractional Na^+^ and K^+^ excretions. Kidneys were processed to obtain samples for immunohistochemistry and Western blotting. All physiological values were normalized by body weight.

### Immunohistochemistry

Immunolocalization studies were performed using an indirect immunoperoxidase technique. Briefly, the renal tissue samples, 3–4 mm thick, were fixed by immersion in Bouin’s solution for 18–24 h at 21–24°C. Then the samples were dehydrated, embedded in Paraplast Plus, sectioned at 5–7 μm thickness with a rotatory microtome (Leica Biosystems, Germany), and mounted on glass slides. Kidney sections were dewaxed, hydrated and washed in Tris–phosphate buffer, pH 7.6 and incubated overnight with anti-COX-2 at 1:500 (SC-1747, Santa Cruz, CA), at room temperature. This was followed by incubation with the corresponding secondary antibody and with the peroxidase-antiperoxidase complex (MP Biomedicals, Santa Ana, CA). Peroxidase activity was detected by incubation of the sections with 0.1% (wt/vol) 3,3′-diaminobenzidine and 0.03% (vol/vol) hydrogen peroxide. Kidney sections were analyzed as previously described (Villanueva et al., 2008) using Nikon Eclipse E600 microscopy and Nikon DS-Ri1 camera (Nikon Corp., Tokyo, Japan). Four representative fields for each section were used and an average of six rats quantified. Images were quantified by Simple PCI 6.0 software (Hamamatsu Corporation, Sewickley, PA) and average values of stained areas were expressed as fold change of controls.

### Biochemical Parameters

Serum and urine electrolytes were assayed using an ion selective electrolyte analyzer model 9180 (Roche Diagnostic, Mannheim, Germany).

### Western Blot

Kidneys were stored at −80°C and then were defrosted. Fifty mg of medullary or cortical kidney tissues were homogenated in lysis RIPA buffer (50 mM Tris-HCl pH 8.0; 150 mM NaCl; 1% NP-40; 0.5% sodium deoxycolate; 0.1% SDS) and centrifuged at 14,000 rpm during 10 min at 4°C. Protein concentrations was determined by Bradford method using Protein Assay (Bio-Rad, Hercules, CA). Total protein content was denatured in SDS-PAGE sample buffer for 20 min at 60°C. To assess consistency of protein loading, 15 µg of protein from each sample were resolved by SDS-PAGE. For immunoblot, each sample was run at 40 µg/lane. Proteins were transferred to PVDF membranes (Bio-Rad, Hercules, CA) blocked with 5% non-fat milk and blotted with each antibody. Primary antibodies used included the following: mouse anti renin/prorenin (B-12 133145, Santa Cruz, CA) at 1:2,000; mouse anti AGT (IBL-20101 OG-922, Japan) at 1:10,000; mouse anti ACE (CD 143, Chemicon Millipore, Warford, UK); rabbit anti ACE2 (H-175 SC-20998, Santa Cruz, CA) at 1:1,000; rabbit anti COX-2 (Cayman 160126, Ann Arbor, MI) at 1:2,000, incubates for 2 h at room temperature. Rabbit anti-cathepsin G antibody (ab197354, Abcam, Cambridge, UK), rabbit anti-chymase (ab233103 Abcam, Cambridge, UK)) and rabbit anti AT1 receptor (sc-515884, Santa Cruz, CA) were used at 1:200 and incubated 24 h at room temperature. Secondary antibodies were tagged with horseradish peroxidase (Santa Cruz, CA, USA). Immunoreactivity was assessed by chemioluminiscence kit (Perkin-Elmer, Life Sciences, Boston, MA, USA). Arbitrary density units were normalized to the mean intensity of control group, defined as 1.0. Values were averaged and mean values compiled for statistical analysis.

### Statistical Analysis

Results are expressed as mean ± SEM. Grubb’s test was used to detect outliers in univariate data assumed to come from a normally distributed population. Comparisons between groups were performed using One-Way ANOVA when appropriate with Tukey’s post-test. P ≤ 0.05 values were considered statistically significant, P = NS is not significant.

## Results

### Potassium Diet Attenuates the Increase in Systolic Blood Pressure in Ang II Infused Rats

**Figure 1:**
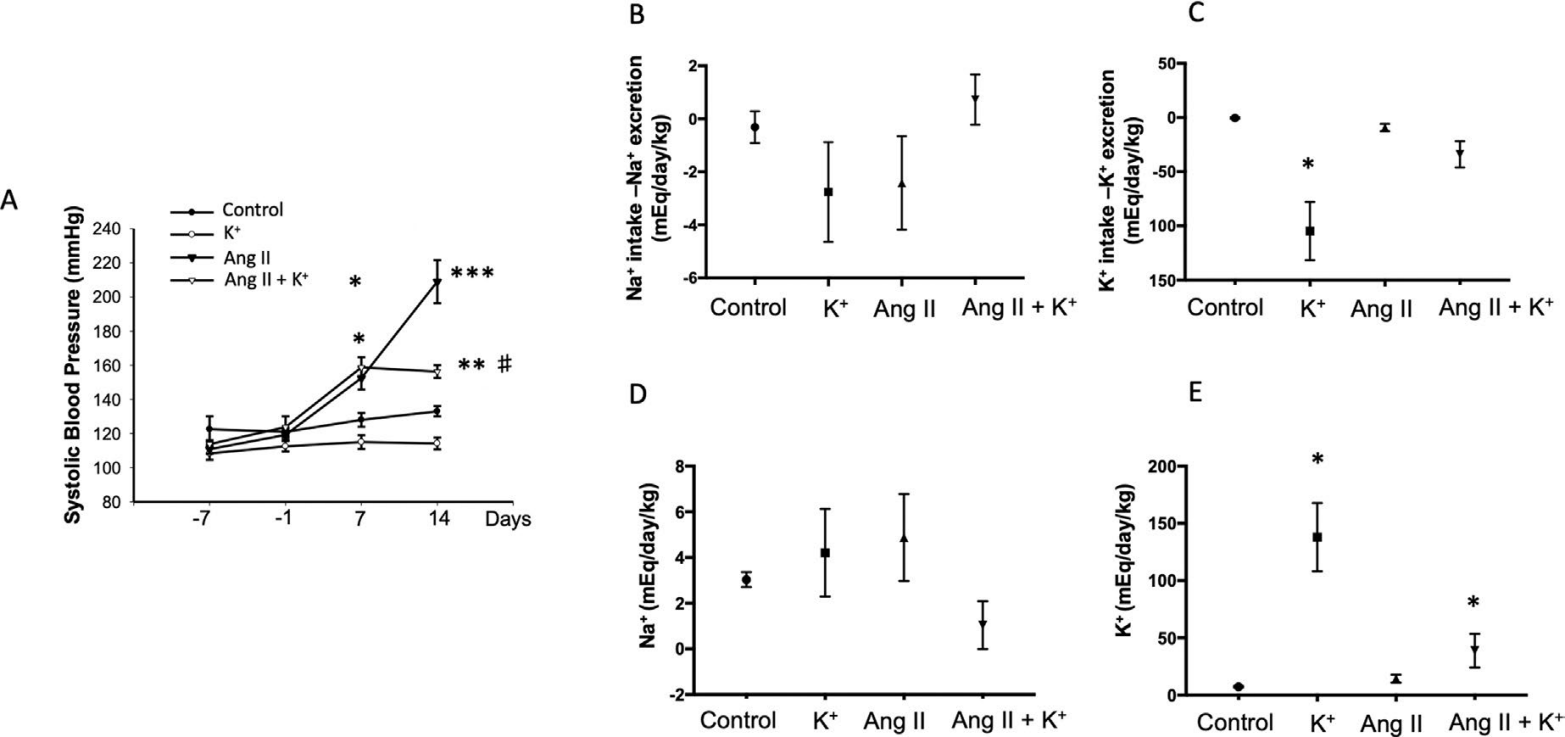
Systolic blood pressure (SBP, tail cuff method) and Na^+^, K^+^ balance in response to chronic Ang II infusion (400 ng/kg/min during 14 days) with or without K^+^ dietary supplementation. **(A)** No changes in basal SBP were observed at day −7. K^+^ dietary supplementation was started at day −7 and SBP was recorded at day 7. After 2 weeks of treatment K^+^ caused a reduction in SBP as compared to controls (black circles), the significant differences were maintained until day 14. SBP was increased by day 7 in Ang II infused rats, as well as in Ang II + K^+^ group. By day 14 Ang II group reached 209 ± 13 mmHg, while Ang II + K^+^ group reached 153 ± 4 mmHg, *P < 0.05, **P < 0.01, ***P < 0.001 vs. control group (normal K^+^ diet, sham operated); ^#^P < 0.05 vs. Ang II group, n = 6. **(B)** Na^+^ and K^+^ **(C)** balance in mEq/day/body mass. **(D)** and **(E)** show U_v_ Na^+^ and K^+^ in mEq/day/kg.

Systolic blood pressure was recorded starting 1 week before minipump implantation. Dietary K^+^ supplementation with 1% KCl started one before Ang II infusion; however, a slight but not significant reduction was observed at day −1. Systolic blood pressure increased in Ang II-infused rats starting at day 7 (153 ± 7 mmHg vs. control group, 128 ± 4 mmHg, P = 0.032) and progressively increased up to 209 ± 13 mmHg vs. 133 ± 3 mmHg, P = 0.0008) by day 14. The Ang II + K^+^ group showed similar increase at day 7 (159 ± 6 mmHg); however, at day 14, the increases in systolic blood pressure were attenuated and significantly reduced as compared to Ang II-infused rats with regular K^+^ diet (156 ± 4 mmHg vs. 209 ± 13 mmHg, P = 0.012, **Figure 1A**).

### Diuretic Responses and Na^+^, K^+^ Balance With High Potassium Diet

Urinary flow was greatly increased in rats supplemented with dietary K^+^ (20.37 ± 2.03 vs. 1.19 ± 0.04 mL/kg/h, P = 0.022). As reported before, Ang II infusion induces pressure diuresis (6.54 ± 1.74 vs. 1.19 ± 0.04 mL/kg/h, P = 0.011), and this effect seems to be enhanced by K^+^ supplementation (8.82 ± 2.52 vs. 1.19 ± 0.04 mL/kg/h, P = 0.010). Sodium balance after 14 days of treatment was close to 0 in control groups, decreased in K^+^ and Ang II groups and slightly decreased in K^+^ group (**Figure 1B**). However, K^+^ balance was reduced in K^+^ group. Combined Ang II infusion plus K^+^ showed no differences with control in K^+^ and Na^+^ balance (**Figures 1B, C**). No differences were found in U_v_ Na^+^ among groups (**Figure 1D**). However, U_V _K^+^ was induced by K^+^ diet as well as by Ang II infusion (**Figure 1E**).

### Potassium Intake Prevented the Induction of AGT, Tubular Renin and ACE in Ang II-Infused Rats

**Figure 2:**
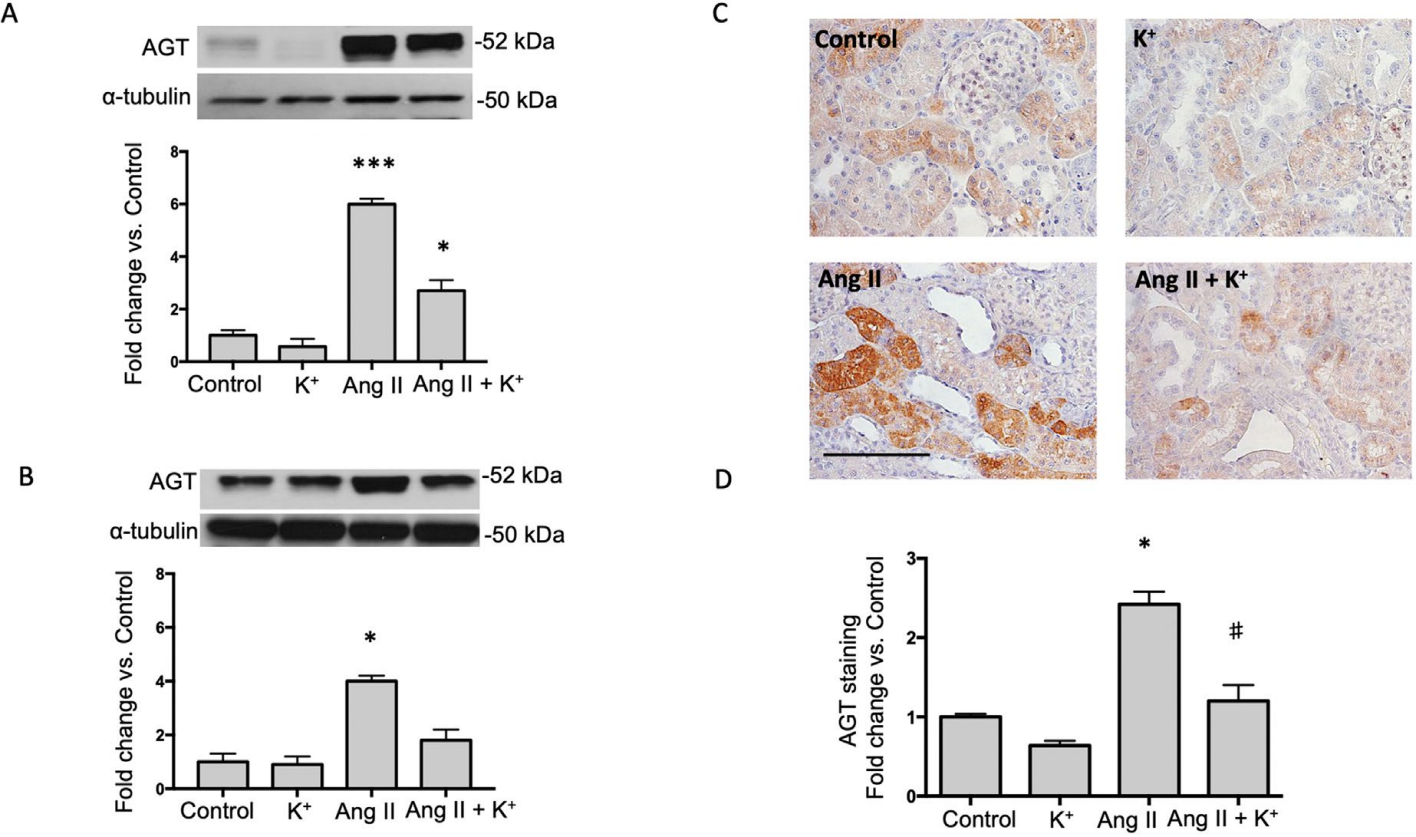
Potassium intake attenuates the induction of angiotensinogen (AGT) expression in Ang II-infused rats. Representative immunoblot and densitometric analysis of AGT protein in renal cortex **(A)** and medulla **(B)** of control rats, K^+^ supplemented, Ang II infused and Ang II-infused + K^+^ supplementation. α-tubulin was used to correct variation in sample loading. Values are expressed as fold change of control group. **(C)** A representative image of AGT immunostaining showing the typically proximal tubule specific staining, which is increased by Ang II infused rats. KCl supplementation prevented this effect. **(D)** Quantification of stained areas in four representative fields for each section from six rats. Images were quantified by Simple PCI 6.0 software and the average values of stained areas were expressed as fold change of controls. *P < 0.05, ***P < 0.001 vs. control group (normal K^+^ diet, sham operated); ^#^P < 0.05 vs. Ang II group, n = 6. Scale bar: 100 µm.

**Figure 3:**
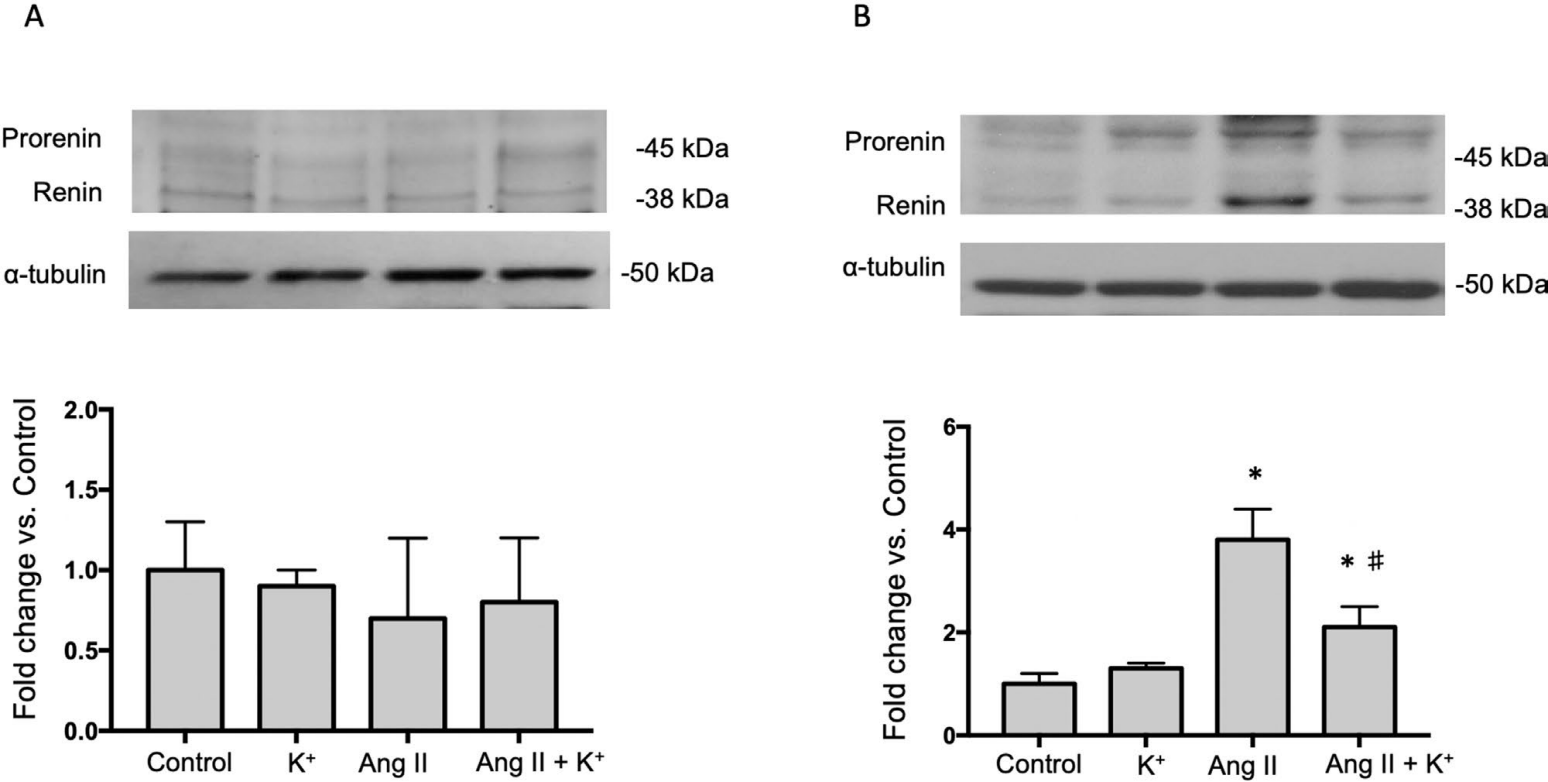
Representative immunoblot and densitometric analysis of prorenin and renin bands in renal cortex **(A)** and medullary tissues **(B)** of control rats, K^+^ supplemented, Ang II infused and Ang II infused + K^+^ dietary supplementation. Ang II infusion enhances prorenin and renin protein abundances in renal medulla and K^+^ intake partially prevented this effect. Micro-dissected inner medullas were used as source of collecting duct renin to avoid juxtaglomerular renin derived. Note that in cortical tissue renin band is more prevalent than prorenin band (data not shown). *P < 0.001 vs. control group (normal K^+^ diet, sham operated); ^#^P < 0.05 vs. Ang II group, n = 6. α-tubulin was used to correct variation in sample loading (same cortical α-tubulin was used for AGT and renin). Values are expressed as fold change of control group.

The expression of AGT protein and mRNA are expressed in proximal tubules and can be detected in cortical renal tissues. We performed semi-quantitative immunoblots using homogenates from micro-dissected cortical tissues of control rats, K^+^ supplemented, Ang II infused + regular K^+^ diet and Ang II + high dietary K^+^ (**Figure 2**). No changes in AGT protein abundances were found in rats with high K^+^ diet vs. those treated with regular diet in the renal cortex (0.6 ± 0.3 vs. 1.0 ± 0.2, P = 0.34, **Figure 1A**) or in the renal medulla (0.9 ± 0.2 vs. 1.0 ± 0.2, P = 0.44, **Figure 1B**). AGT protein abundance was increased in Ang II-infused rats in the renal cortex (6.0 ± 0.2 vs. 1.0 ± 0.2, P = 0.0003). K^+^ supplementation partially prevented the induction of AGT in Ang II-infused rats (2.7 ± 0.4 vs. 6.0 ± 0.2, P = 0.033). Similar results were observed by analyzing the stained area of cortical AGT showing no changes with K^+^ diet (0.64 ± 0.06 vs. 1.00 ± 0.04, P = 0.48) but increased staining in the Ang II group (2.42 ± 0.16 vs. 1.00 ± 0.04, P = 0.031). As observed in Western blot results, Ang II + K^+^ prevented AGT induction when compared to Ang II rats (1.17 ± 0.13 vs. 2.42 ± 0.16, P = 0.013), see **Figures 2C, D**. Prorenin and renin are expressed in the CD, and their abundances are increased in chronic Ang II-infused rats (Prieto-Carrasquero et al., 2004). To avoid contamination of the juxtaglomerular renin component, we used micro-dissected tissues exclusively from renal inner medullary tissues to measure renin as source of CD. Prorenin plus renin protein levels did not show changes in the renal cortex (**Figure 3A**), however, both bands were augmented in medullary tissues of Ang II-infused rats compared to control group (3.8 ± 0.6 vs. 1.0 ± 0.2, P < 0.0009). Although K^+^ supplementation increases renin plus prorenin protein expression in Ang II-infused rats (2.1 +/- 0.4 vs. 1.0 ± 0.2, P < 0.033), it was significantly less that of the Ang II group (2.1 ± 0.4 vs. 3.8 ± 0.6, P = 0.011, (**Figure 3B**).

### Potassium Intake Prevents Ang II-Dependent Upregulation of ACE and Increases the Expression of ACE2 in the Renal Medulla

**Figure 4:**
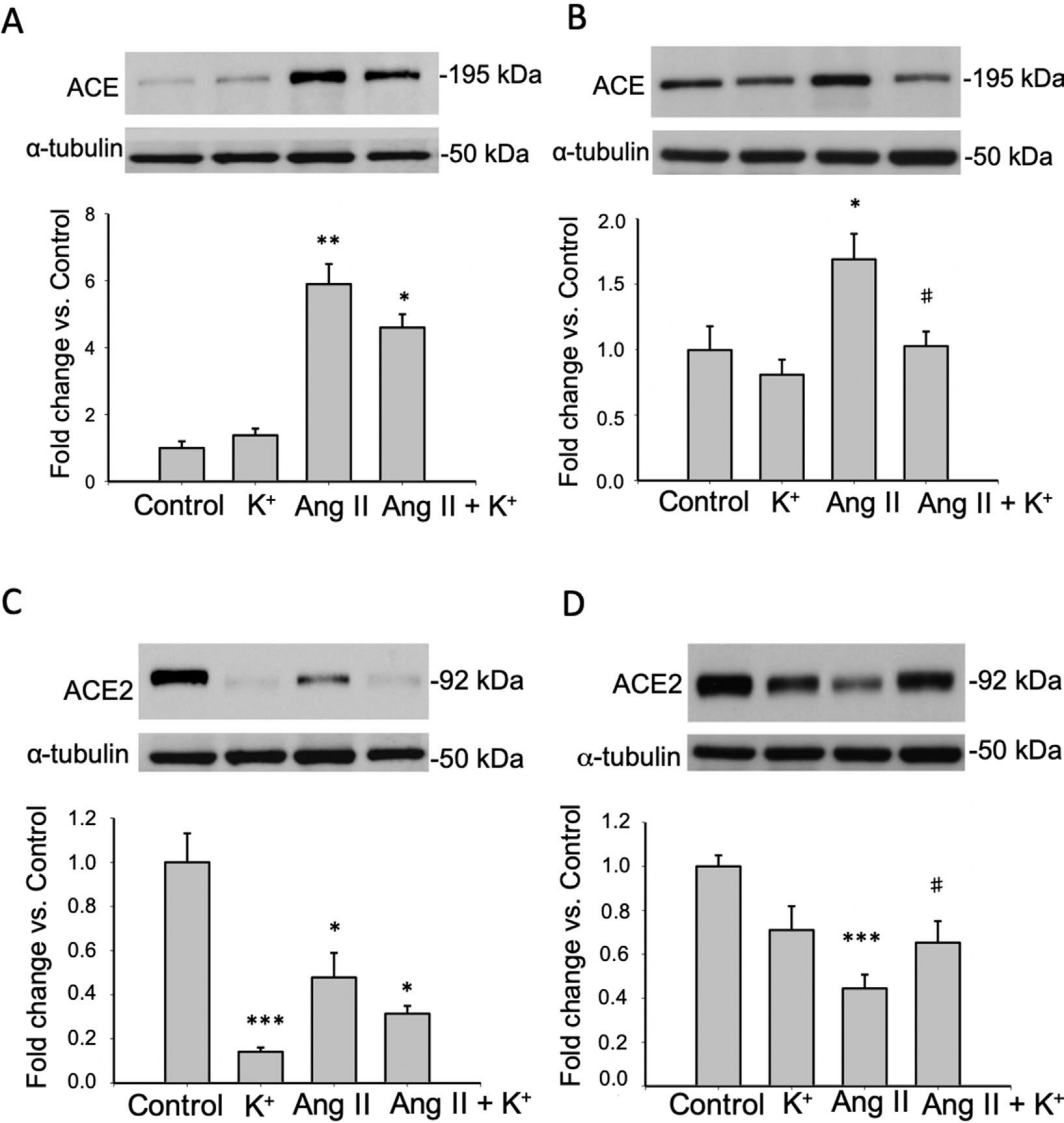
Reciprocal changes in ACE and ACE2 in response to K^+^ intake in rats infused with Ang II. ACE protein expression is shown in cortex **(A)** and medulla **(B)**. ACE2 protein abundances are shown in cortex **(C)** and medulla **(D)**. *P < 0.05, **P < 0.01, ***P < 0.001 vs. control group, ^#^P < 0.05 vs. Ang II group, n = 6. α-tubulin was used to correct variation in sample loading. Values are expressed as fold change of control group.

AGT can be cleaved by CD renin to form Ang I. It is expected that ACE expressed in the luminal side of the proximal tubule or CD promote its conversion to Ang II. We analyzed the expression of ACE in both, cortical and medullary tissues and compared them to control tissues as a fold change defined as 1.0 in controls. K^+^ dietary supplementation did not alter ACE expression in renal cortical (**Figure 4A**) or medullary (**Figure 4B**) renal tissues. ACE protein levels were augmented in cortical (5.87 ± 1.10 vs. 1.00 ± 0.21, P = 0.008) and medullary tissues (1.68 ± 0.19 vs. 1.00 ± 0.18, P = 0.009) of Ang II infused rats. K^+^ dietary supplementation completely prevented the induction of ACE in the renal medulla (1.02 ± 0.11 vs. 1.00 ± 0.18, P = 0.89) but not in the cortex (4.63 ± 0.54 vs. 1.00 ± 0.21, P = 0.0084). We next investigated the effect of Ang II infusions and K^+^ diet on ACE2 expression in cortical and medullary tissues. As shown in **Figures 4C, D**, K^+^ diet causes a reduction in ACE2 expression in normotensive rats (non-infused), this effect was evident in the cortex (0.14 ± 0.01 vs. 1.00 ± 0.13, P = 0.0004) but not in the medulla (0.71 ± 0.11 vs. 1.00 ± 0.14, P = 0.34). In Ang II infused + normal K^+^ diet ACE2 was also reduced in both the cortex and in the medulla. The reduction of ACE2 protein expression caused by Ang II infusion in medullary tissues (Ang II: 0.44 ± 0.06, P = 0.00023 vs. control) was partially reversed in the Ang II + K^+^ diet (0.65 ± 0.09, P = 0.08 vs. control).

### COX-2 Protein Expression is Induced by Dietary K^+^ Intake in the Renal Medulla

**Figure 5:**
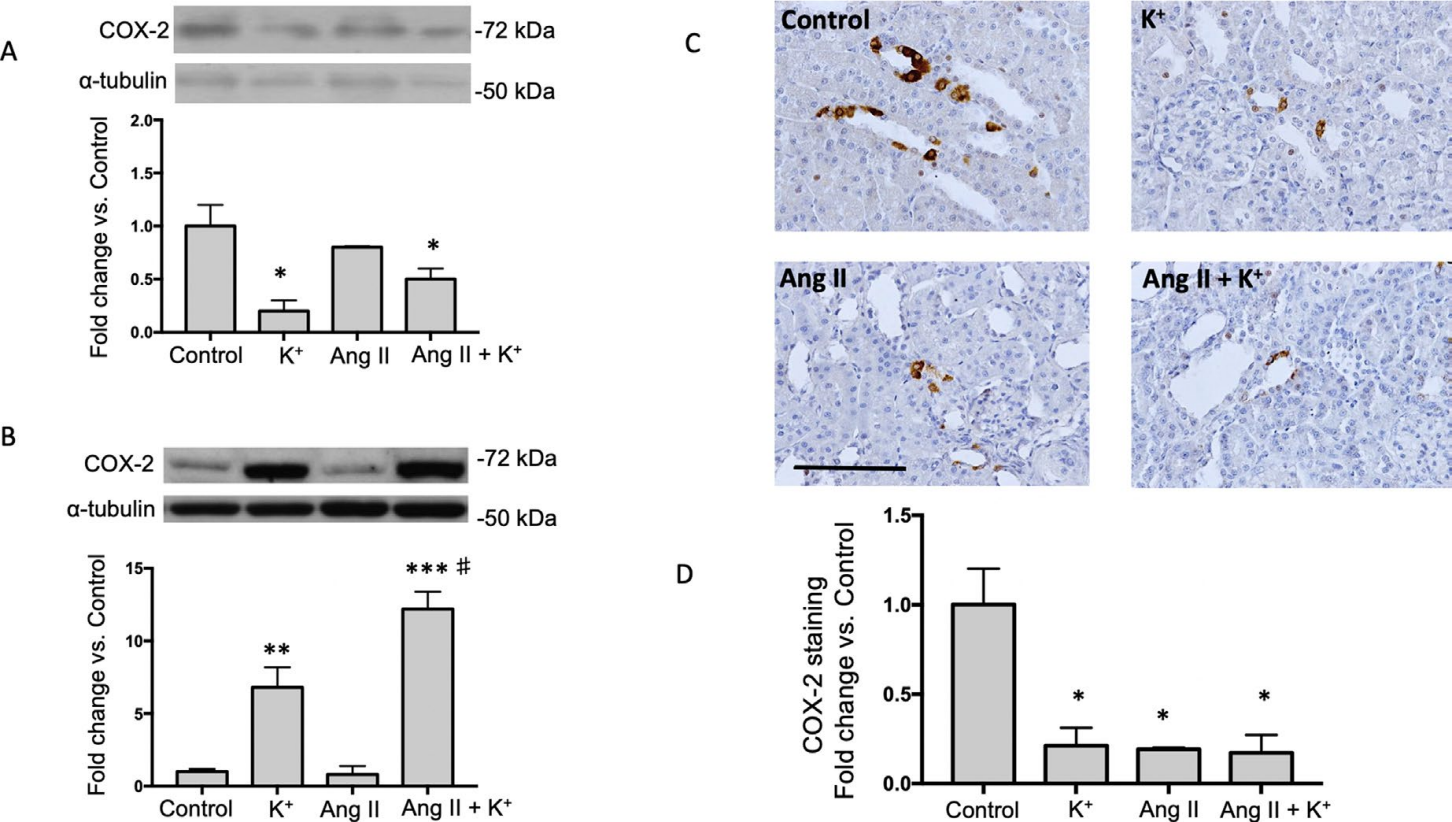
COX-2 is induced in the renal medulla of non-infused and Ang II infused rats supplemented with high K^+^ diet but not in cortex. Representative immunoblot and densitometric analysis showing fold change vs. controls in cortex **(A)** and medulla **(B)**. Representative images of immunohistochemistry showing positive cells (brown color) for COX-2 in **(C)** **(D)** Quantification of stained areas in four representative fields for each section from six rats. Images were quantified by Simple PCI 6.0 software and the average values of stained areas were expressed as fold change of controls. *P < 0.05, **P < 0.01, ***P < 0.001 vs. control group (normal K^+^ diet, sham operated); ^#^P < 0.05 vs. Ang II group, n = 6. α-tubulin was used to correct variation in sample loading. Values are expressed as fold change of control group. Scale bar: 100 µm.

Because activation of bradykinin B2 receptor enhances COX-2 in the kidney, we investigated if high K^+^ diet might involve the induction of medullary COX-2. In the renal medulla COX-2 mediated PGE_2_ production stimulates vasodilation and natriuresis (Gonzalez et al., 2009) contributing to the benefic effects of K^+^ diet. As shown in **Figure 5A**, K^+^ diet downregulates cortical COX-2 (0.23 ± 0.09 vs. 1.00 ± 0.21, P = 0.007), but greatly induces medullary COX-2 (6.8 ± 1.4 vs. 1.0 ± 0.2, P = 0.007, **Figure 5B**). No additional changes of COX-2 expression were detected in cortical COX-2. Ang II infusions did not change COX-2 expression (0.8 ± 0.6-fold change of control, P = 0.42). However, Ang II infused rats with high K^+^ diet showed a 12-fold increase in COX-2 expression in medullary tissues (12.2 ± 1.1 vs. 1.0 ± 0.2, P < 0.001). COX-2 immunostainings showed a significant decrease in rats with K^+^ diet (0.29 ± 0.04, P = 0.0033), Ang II (0.26 ± 0.04, P = 0.0024) and Ang II + K^+^ group (0.23 ± 0.02, P = 0.010) when compared to control rats (1.00 ± 0.19), see **Figures 5C, D**.

### Potassium Intake Increases Kallikrein Protein Expression in Normotensive and in Ang II-Dependent Hypertensive Rats

**Figure 6:**
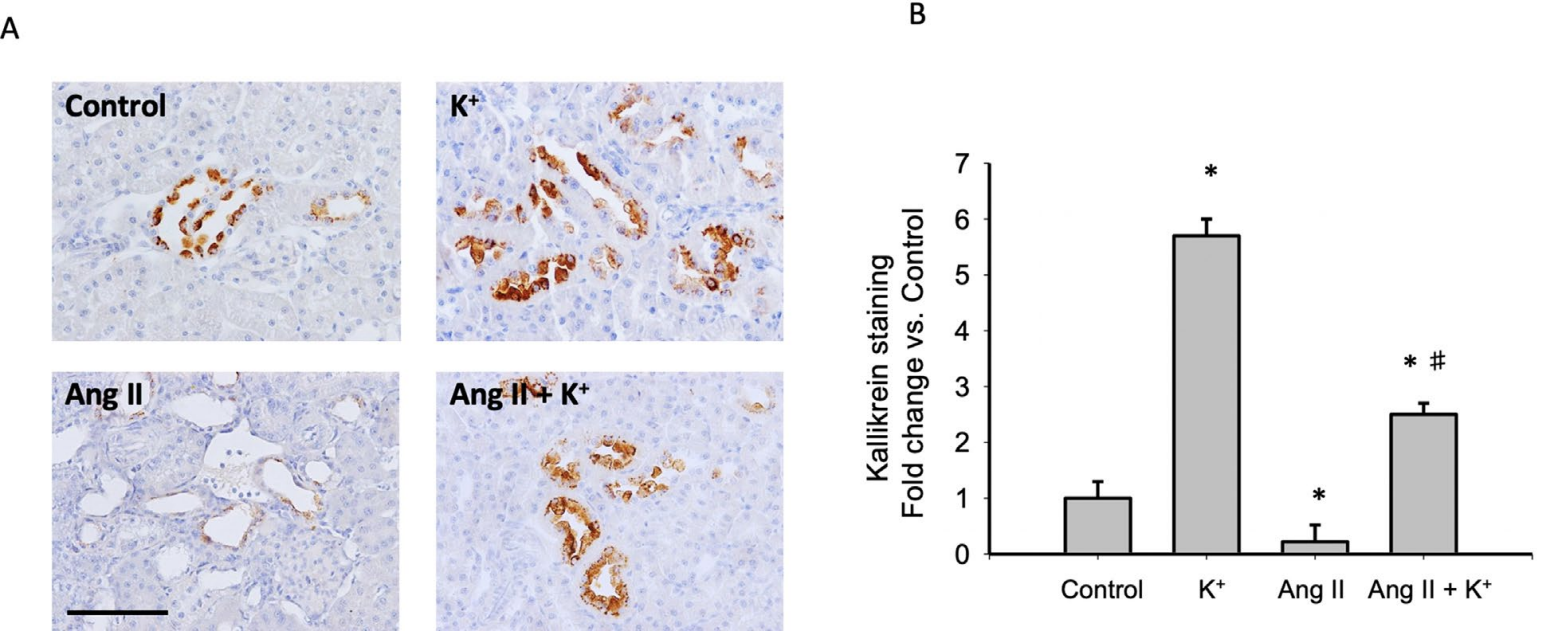
**(A)** Representative images of immunohistochemistry showing positive cells (brown color) for kallikrein protein. **(B)** Quantification of stained areas in four representative fields for each section from six rats. Images were quantified by Simple PCI 6.0 software and the average values of stained areas were expressed as fold change of controls. *P < 0.05 vs. control group (normal K^+^ diet, sham operated); ^#^P < 0.05 vs. Ang II group, n = 6. Scale bar: 100 µm.

To explore the effect of high K^+^ diet on kallikrein expression we performed immunolocalization studies using an indirect immunoperoxidase technique in kidney slides. **Figure 6A** shows representative images of all groups while **Figure 6B** shows the quantification expressed as fold change of control (normal K^+^ diet non infused-sham operated). As observed, K^+^ caused a sixfold increase in the staining area as compared to controls, while Ang II infusion dramatically reduced kallikrein staining (0.19 ± 0.02, P = 0.02 vs. control). Importantly, K^+^ diet reversed this effect increasing kallikrein expression (2.5 ± 0.2 vs. 1.0 ± 0.3, P = 0.0031).

### Expression of Chymase and Cathepsin G During High Potassium Intake in Normotensive and in Ang II-Dependent Hypertensive Rats

**Figure 7:**
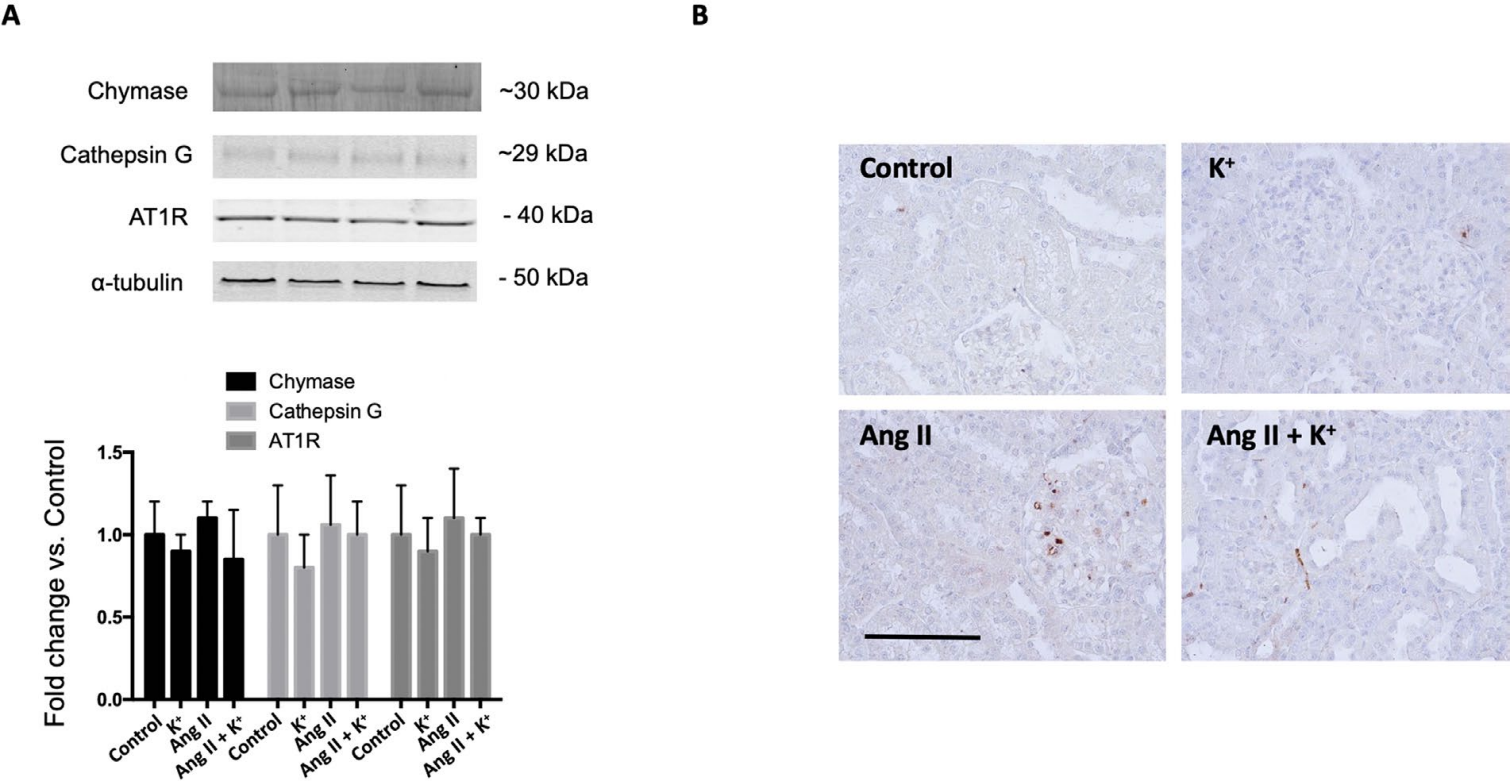
**(A)** Expression of chymase, cathepsin G and the angiotensin II type 1 receptor (AT1) during high potassium intake in normotensive and Ang II-dependent hypertensive rats assessed by immunoblot. No significant changes were observed in protein abundances. **(B)** Immunohistochemical detection of chymase. Scale bar: 100 µm, n = 6.

Since it has been described that the kidney expresses alternative Ang II-producing enzymes such as chymase and cathepsin G, we evaluated protein levels of these two enzymes by immunoblot (**Figure 7A**) upon Ang II and K^+^ treatments. We did not detect changes in protein levels by immunoblots. Immunohistochemistry images showed a few chymase positive cells and did not show significant changes in cell number or intensity (data not shown) (**Figure 7B**).

## Discussion

In this study we demonstrated that Ang II-dependent blood pressure responses at day 14 of treatment are partially prevented by dietary supplementation of K^+^. We also showed that Ang II-dependent induction of CD renin, AGT and ACE was prevented by K^+^ diet. Dietary K^+^ supplementation also increased the expression of ACE2 and COX-2 in the renal medulla. This was paralleled with enhanced K^+^ excretion. Pressure diuresis observed in Ang II-infused rats was slightly but non-significantly increased by high K^+^ diet. Similarly, urine flow was augmented in normotensive (non-infused) and in Ang II-dependent hypertensive rats with high K^+^ diet. These findings indicate that the reciprocal changes caused by high K^+^ in intrarenal RAS and in medullary COX-2 and ACE2 promote vasodilation and diuretic effects, buffering the effects of Ang II.

Induction of intrarenal RAS in the Ang II-dependent hypertensive model has been widely reported in rats (Yang and Xu, 2017). Expression of AGT has been described in proximal tubules and its synthesis and secretion to luminal fluid is stimulated by chronic Ang II *via* the AT1R mechanism (Kobori et al., 2001). AGT can be detected in urine samples of hypertensive humans (Kobori et al., 2009) and in animal models of hypertension (Gonzalez-Villalobos et al., 2008) and renal disease (Kobori et al., 2003). AGT is the only source for renin enzyme to produce Ang I (Chaszczewska-Markowska et al., 2016). Cumulated evidence demonstrates that renin expression is enhanced in the CD during chronic Ang II infusion (Prieto-Carrasquero et al., 2004) and diabetic disease (Kang et al., 2008). CD renin is regulated by activation of AT1R and independently of blood pressure (Prieto-Carrasquero et al., 2008). Its cardinal role in the development of hypertension is evidenced by the increases in blood pressure in mice with renin overexpression (Ramkumar et al., 2013). Expression of ACE is induced along with the induction of renin synthesis in the CD during Ang II-dependent hypertension (Gonzalez-Villalobos et al., 2010). Knockout models of renal ACE, have demonstrated the absence of hypertension in response to chronic Ang II infusions (Gonzalez-Villalobos et al., 2013). Thus, intrarenal Ang II formation stimulated by the induction of AGT, renin and ACE may contribute to sodium and water retention and high blood pressure. Here we show that induction of AGT and collecting duct renin from Ang II infused rats is prevented by K^+^ dietary supplementation. Although AGT was still high in the cortex the induction was less evident (**Figure 2**). It is possible that the reduced expression of intrarenal RAS components may impact the availability of the distal nephron to produce intratubular Ang II. Ang II causes aldosterone release from adrenals and mineralocorticoid receptor (MR)-mediated Na^+^ reabsorption through ENaC in the CD (Beutler et al., 2003). In addition, AT1R in the apical side of the CD cells (Harrison-Bernard et al., 2002) directly stimulates the activity of ENaC leading to increased sodium reabsorption (Mamenko et al., 2013). Furthermore, treatment with amiloride (ENaC inhibitor) reduces blood pressure in Ang II infused rats (Gonzalez et al., 2011). This suggests a major role of luminal Ang II and apical AT1R in the stimulation of apical sodium transport in connecting tubules and in the CD. Indeed, it has been shown that K^+^ decreased the abundance of NCC expression (Wang et al., 2018). Van der Lubbe et al. showed that K^+^-induced natriuresis was accompanied by inhibition of the Na^+^-Cl^-^ cotransporter (van der Lubbe et al., 2013). Although we did not explore the exact mechanisms by which K^+^ diet decreases intrarenal RAS components, we believe that the absence of intraluminal Ang II formation contributes to lower Ang II-dependent activation of distal sodium transporters. Proteinases such as chymase, and cathepsin G, have been demonstrated to be an alternative pathway for Ang II formation in renal and in heart tissues (DellÌtalia et al., 2018). We observed that the expression of chymase was slightly but not significantly reduced by K^+^ diet or by Ang II infusion. The same pattern was observed for cathepsin G (**Figure 7A**). We were unable to detect cathepsin G by using immunohistochemistry. We detected a few cells stained for chymase, mostly in interstitial cells; however, no significant changes in the intensity of labeling or cell number was evidenced (**Figure 7B**). An increase in Ang II levels might not lead to a significant effect if the AT1 receptor is already fully expressed. Our results show a positive correlation between renin and AGT expression which are supported by the atlas of tissue RAS created by (Nehme et al. 2015) This fact, along with the evidence of the high expression of AT1R in the kidneys (Nehme et al. 2019) may eventually lead to a bottleneck if the AT1 receptor is not altered. However, evidence shows that AT1 receptor expression is not modified by Ang II induced hypertension (Harrison et al. 1997). We did not find changes in AT1 receptor expression (**Figure 7A**); thus, we believe that changes in intratubular Ang II instead of changes in AT1 receptor levels are responsible for intratubular actions of Ang II.

Despite these observations, the natriuretic effect seen by the balance between Na^+^ intake and excretion was only partially stimulated by K^+^ diet. This was also similar to the pressure natriuretic effect observed in Ang II infused rats (**Figure 1B**). Dietary K^+^ caused an increased K^+^ excretion with a negative balance between K^+^ intake and excretion, and Ang II infused animals did not show altered K^+^ excretion. Interestingly, Ang II + K^+^ group showed a significant K^+^ excretion as compared to control group.

**Figure 8:**
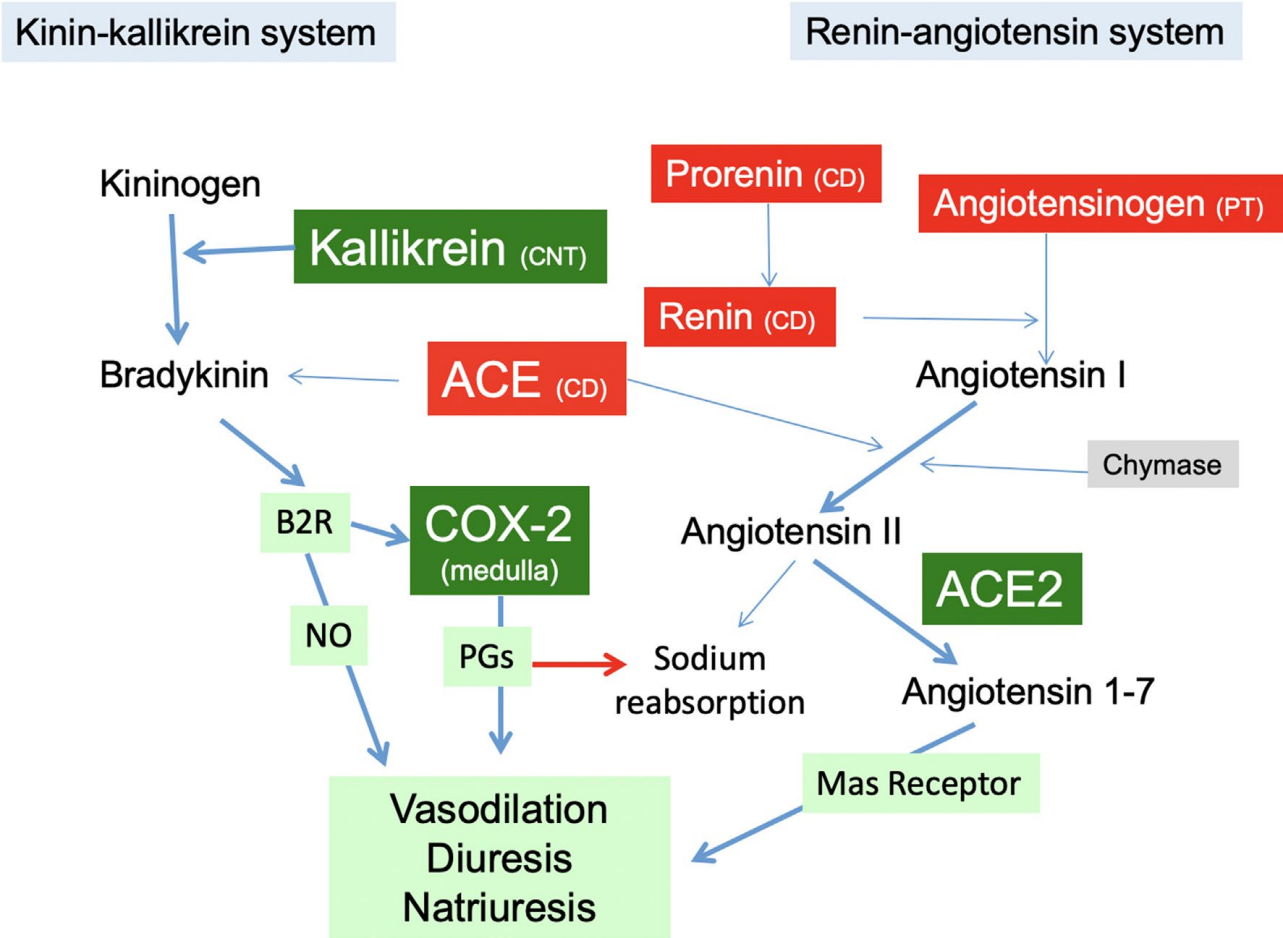
The RAS in the kidney in Ang II-infused rats with high K^+^ diet. Green boxes represent proteins induced by high K^+^ intake in Ang II infused rats (kallikrein, COX-2, ACE2) promoting vasodilatory, diuretic and natriuretic actions through PGs. High K^+^ diet impairs upregulation of the components of the intrarenal RAS (red boxes), reducing the capacity for intratubular Ang II formation. PT, proximal tubule; CD, collecting duct; CNT, connecting tubule; NO, nitric oxide; B2R, bradykinin B2 receptor; COX-2, cyclooxygenase-2; ACE, angiotensin converting enzyme.

It has been shown that K^+^ diet increased the expression of kallikrein, in the connecting tubule41. Kallikrein cleaves kininogen to form bradykinin, which in turn binds to bradykinin B2 receptor (Rodriguez et al. 2004). ACE inhibition also potentiates bradykinin effects (Kaplan et al. 2002), thus the decrease in ACE expression may explain the increase in the kinin-kallikrein system. Activation of the B2 receptor induces the synthesis of COX-2. Our findings demonstrate that K^+^ diet induces the expression of kallikrein which is mainly expressed in connecting tubules (Kaplan et al. 2002) as well as COX-2 expression in the renal medulla of normotensive and Ang II-dependent hypertensive rats. COX-2 induction promotes synthesis of vasodilatory PGs, which may cross the interstitial tissue reaching intratubular fluid to decrease the activity and expression of ENaC (Gonzalez et al., 2009) thereby reducing sodium reabsorption and contributing to lower blood pressure (**Figure 8**).

A recent meta-analyses study of 22 randomized controlled trials in adults demonstrated that increased K^+^ intake reduces systolic blood pressure (Aburto et al. 2013). Systematic review of the literature also showed that increased K^+^ has no adverse effect on blood lipid concentrations, catecholamine concentrations, or renal function. Furthermore, higher K^+^ intake lowers the risk of incident of stroke (Aburto et al. 2013). It is likely that the beneficial effects of high K^+^ diet might be due to the modulation of the expression of the intrarenal RAS and the enhanced expression of peptides with vasodilator effects. Our data indicate that high K^+^ diet causes reciprocal changes in the regulation of vasoactive hormones through the upregulation of natriuretic COX-2, ACE2 and by preventing the upregulation of intratubular/intrarenal RAS during Ang II-dependent hypertension.

## Data Availability Statement

The raw data supporting the conclusions of this manuscript will be made available by the authors, without undue reservation, to any qualified researcher.

## Ethics Statement

The animal study was reviewed and approved by Animal Care and Use Committee of Pontificia Universidad Católica de Chile (Animal Welfare Assurance no. A5848-01) and conducted in accordance with the National Institutes of Health Guide for the Care and Use of Laboratory Animals.

## Author Contributions

AG and CV designed the experiments. CC and MG conducted most of the experiments, collected and analyzed the data. AG and CC wrote the manuscript, and CV contributed to its final revision.

## Funding

This work was supported by: Proyecto de Financiamiento Basal, AFB 170005 and Fondo Nacional de Investigación Científica y Tecnológica FONDECYT 1130741 (CV) and 1191006 (AG).

## Conflict of Interest

The authors declare that the research was conducted in the absence of any commercial or financial relationships that could be construed as a potential conflict of interest.
