# Supplementary material for: Potassium Intake Prevents the Induction of the Renin-Angiotensin System and Increases Medullary ACE2 and COX-2 in the Kidneys of Angiotensin II-Dependent Hypertensive Rats
Source: Front Pharmacol. 2019 Oct 15;10:1212. doi: 10.3389/fphar.2019.01212 (PMC6804396; doi:10.3389/fphar.2019.01212)
Supplement: Supplementary file 1 [file Presentation_1.pptx]

## Slide 1
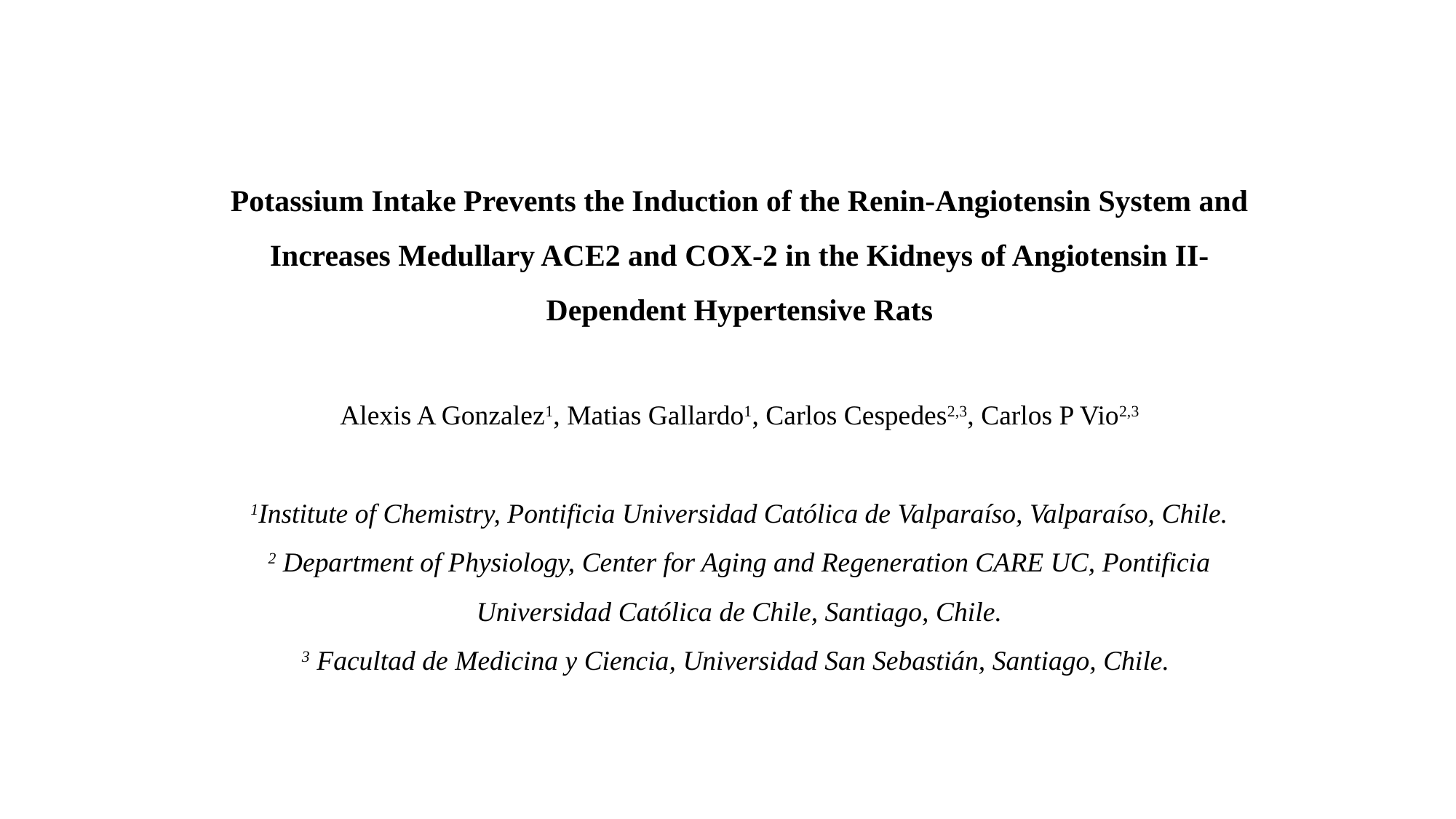

Potassium Intake Prevents the Induction of the Renin-Angiotensin System and Increases Medullary ACE2 and COX-2 in the Kidneys of Angiotensin II-Dependent Hypertensive Rats
Alexis A Gonzalez1, Matias Gallardo1, Carlos Cespedes2,3, Carlos P Vio2,3
1Institute of Chemistry, Pontificia Universidad Católica de Valparaíso, Valparaíso, Chile.
2 Department of Physiology, Center for Aging and Regeneration CARE UC, Pontificia Universidad Católica de Chile, Santiago, Chile.
3 Facultad de Medicina y Ciencia, Universidad San Sebastián, Santiago, Chile.

## Slide 2
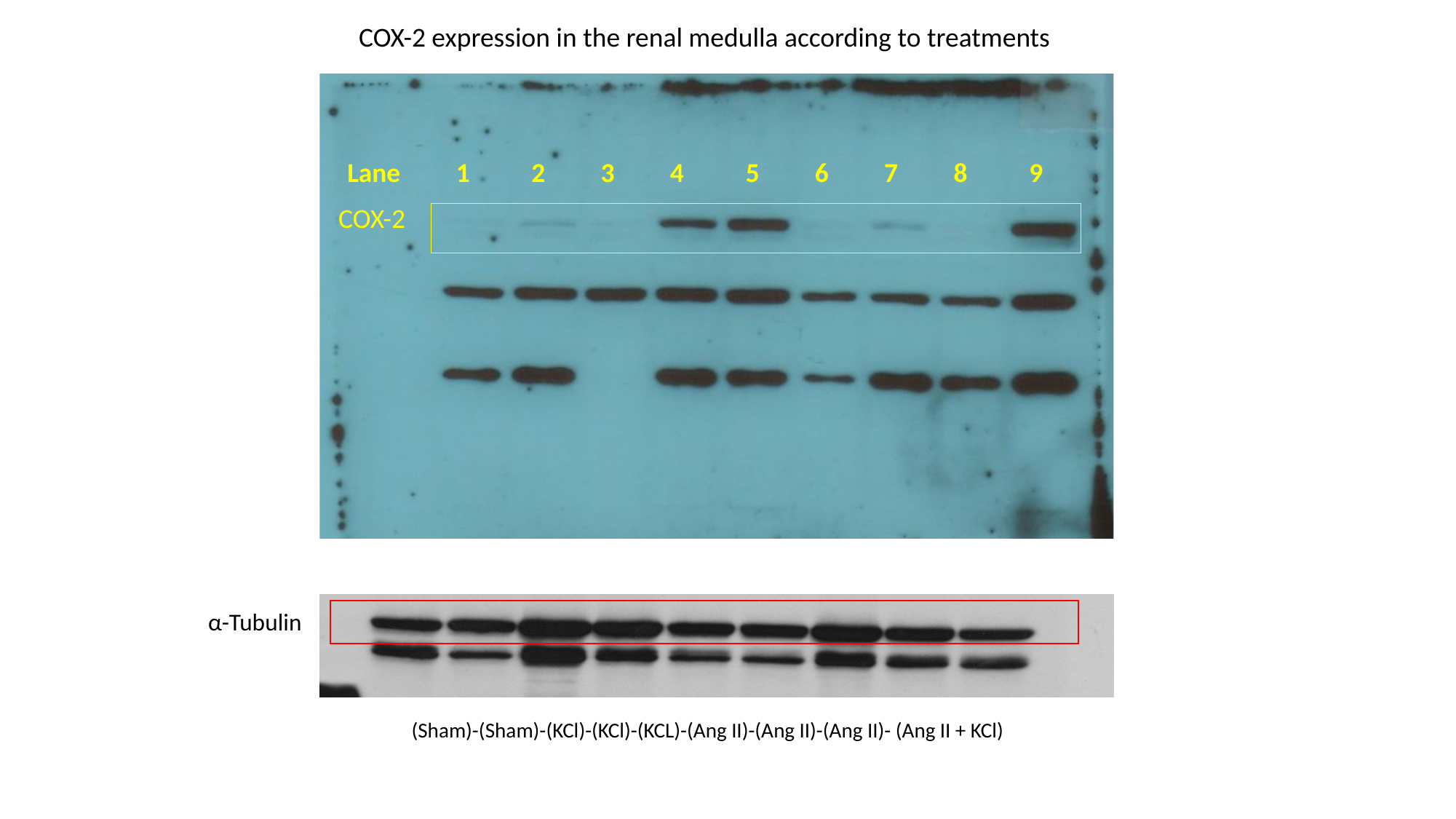

COX-2 expression in the renal medulla according to treatments
 Lane 1 2 3 4 5 6 7 8 9
COX-2
α-Tubulin
 (Sham)-(Sham)-(KCl)-(KCl)-(KCL)-(Ang II)-(Ang II)-(Ang II)- (Ang II + KCl)

## Slide 3
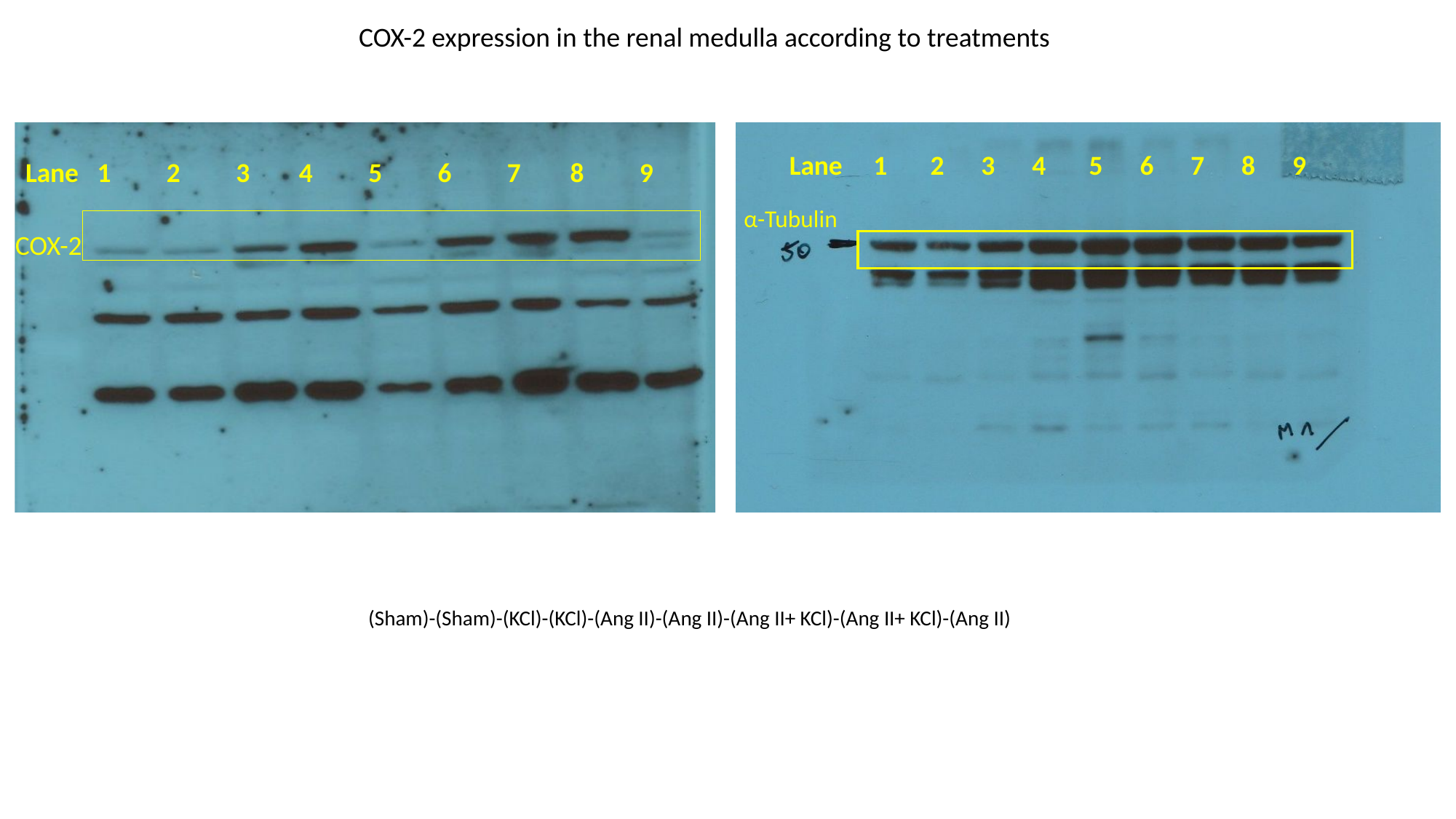

COX-2 expression in the renal medulla according to treatments
Lane 1 2 3 4 5 6 7 8 9
Lane 1 2 3 4 5 6 7 8 9
α-Tubulin
COX-2
(Sham)-(Sham)-(KCl)-(KCl)-(Ang II)-(Ang II)-(Ang II+ KCl)-(Ang II+ KCl)-(Ang II)

## Slide 4
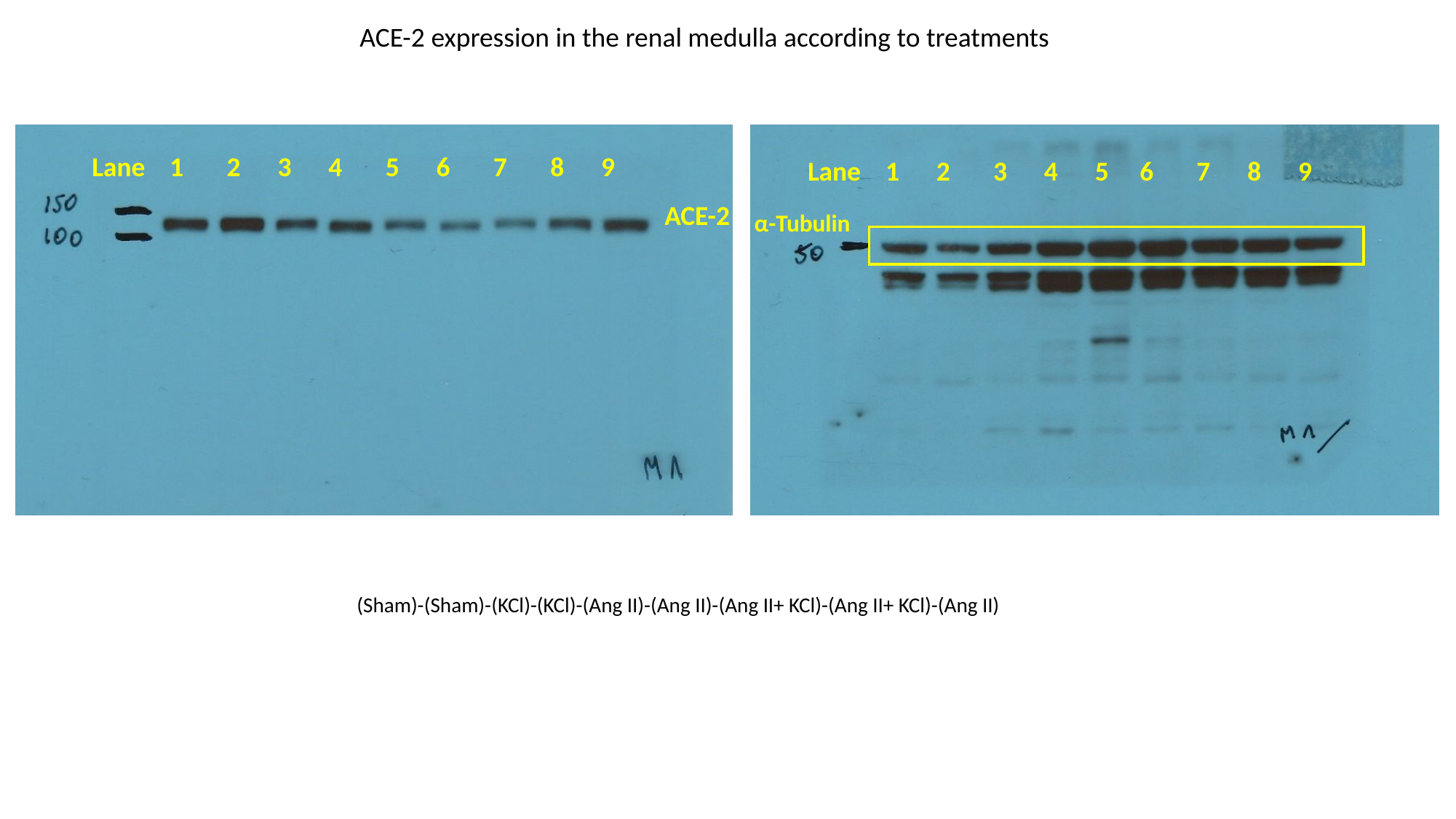

ACE-2 expression in the renal medulla according to treatments
Lane 1 2 3 4 5 6 7 8 9
Lane 1 2 3 4 5 6 7 8 9
ACE-2
α-Tubulin
(Sham)-(Sham)-(KCl)-(KCl)-(Ang II)-(Ang II)-(Ang II+ KCl)-(Ang II+ KCl)-(Ang II)

## Slide 5
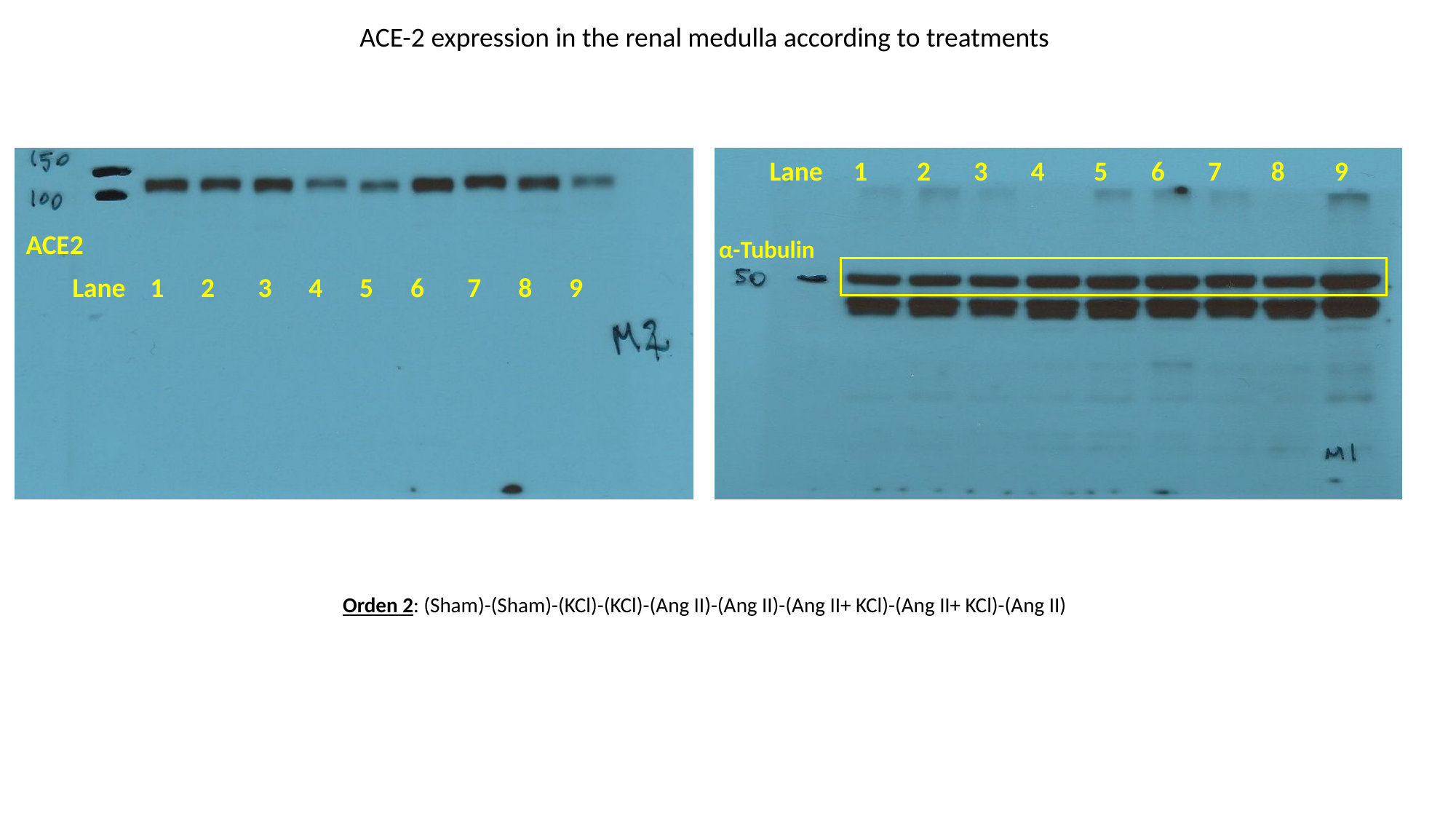

ACE-2 expression in the renal medulla according to treatments
Lane 1 2 3 4 5 6 7 8 9
ACE2
α-Tubulin
Lane 1 2 3 4 5 6 7 8 9
Orden 2: (Sham)-(Sham)-(KCl)-(KCl)-(Ang II)-(Ang II)-(Ang II+ KCl)-(Ang II+ KCl)-(Ang II)

## Slide 6
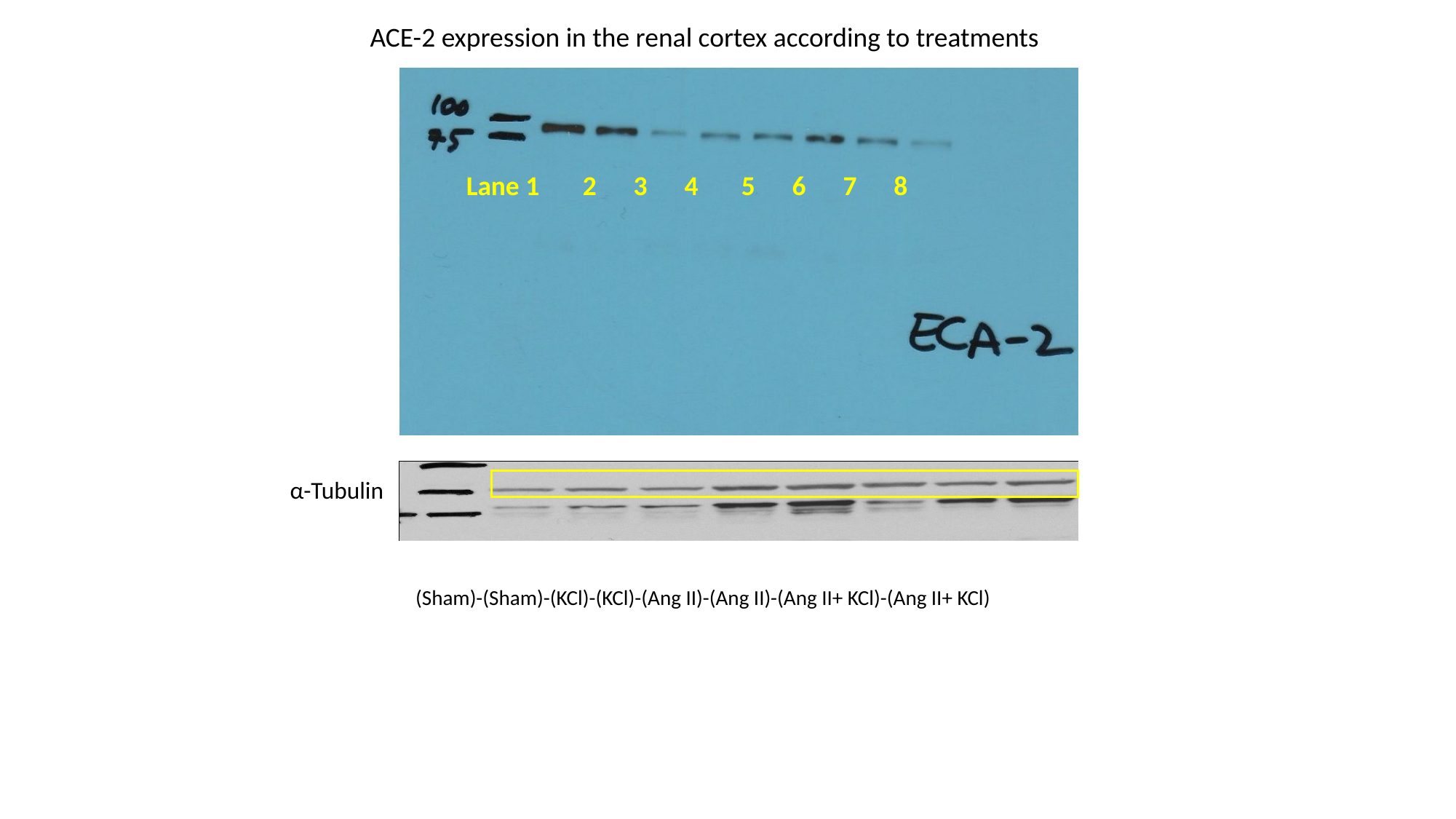

ACE-2 expression in the renal cortex according to treatments
Lane 1 2 3 4 5 6 7 8
α-Tubulin
(Sham)-(Sham)-(KCl)-(KCl)-(Ang II)-(Ang II)-(Ang II+ KCl)-(Ang II+ KCl)

## Slide 7
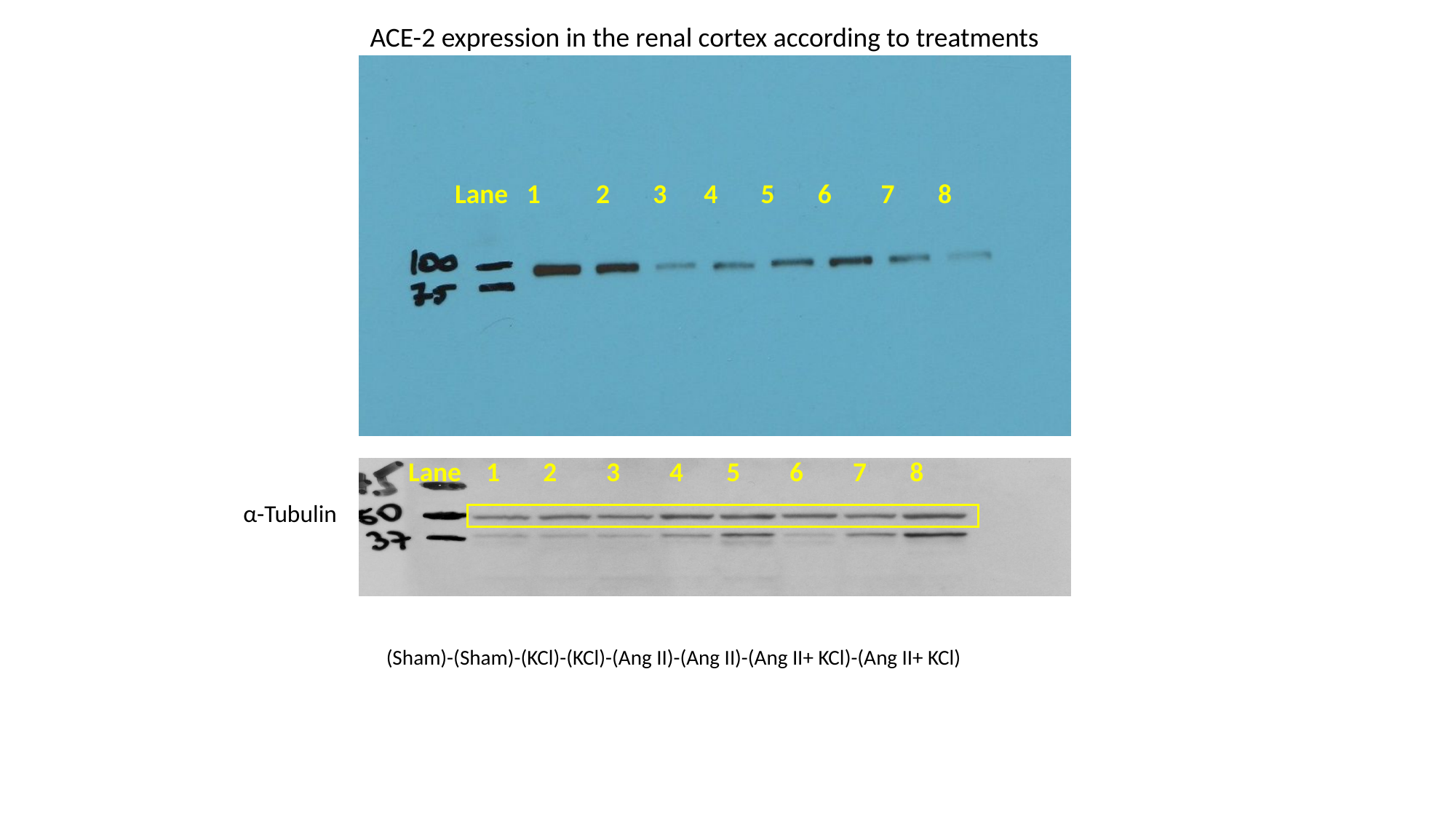

ACE-2 expression in the renal cortex according to treatments
Lane 1 2 3 4 5 6 7 8
Lane 1 2 3 4 5 6 7 8
α-Tubulin
(Sham)-(Sham)-(KCl)-(KCl)-(Ang II)-(Ang II)-(Ang II+ KCl)-(Ang II+ KCl)

## Slide 8
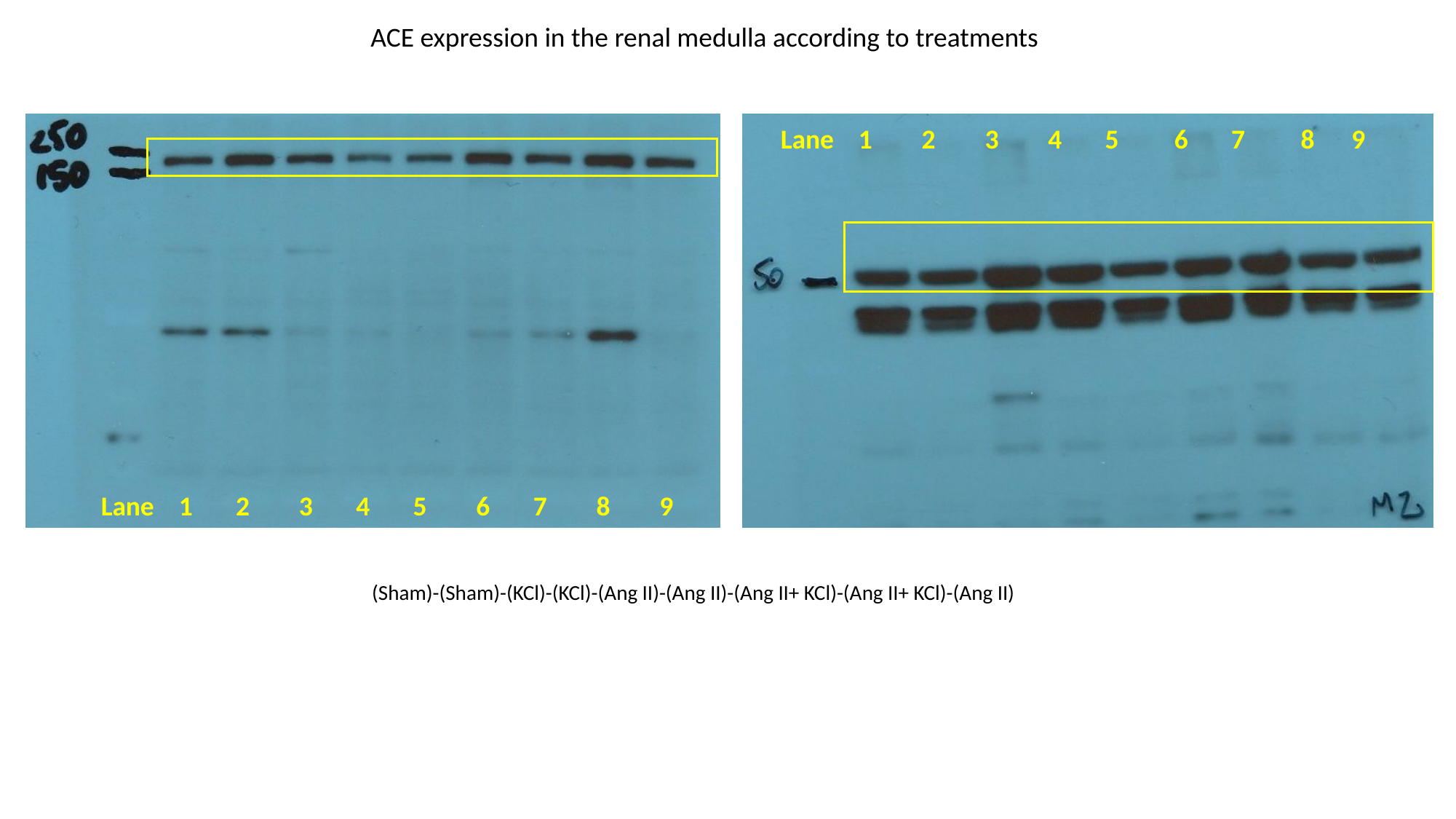

ACE expression in the renal medulla according to treatments
Lane 1 2 3 4 5 6 7 8 9
Lane 1 2 3 4 5 6 7 8 9
(Sham)-(Sham)-(KCl)-(KCl)-(Ang II)-(Ang II)-(Ang II+ KCl)-(Ang II+ KCl)-(Ang II)

## Slide 9
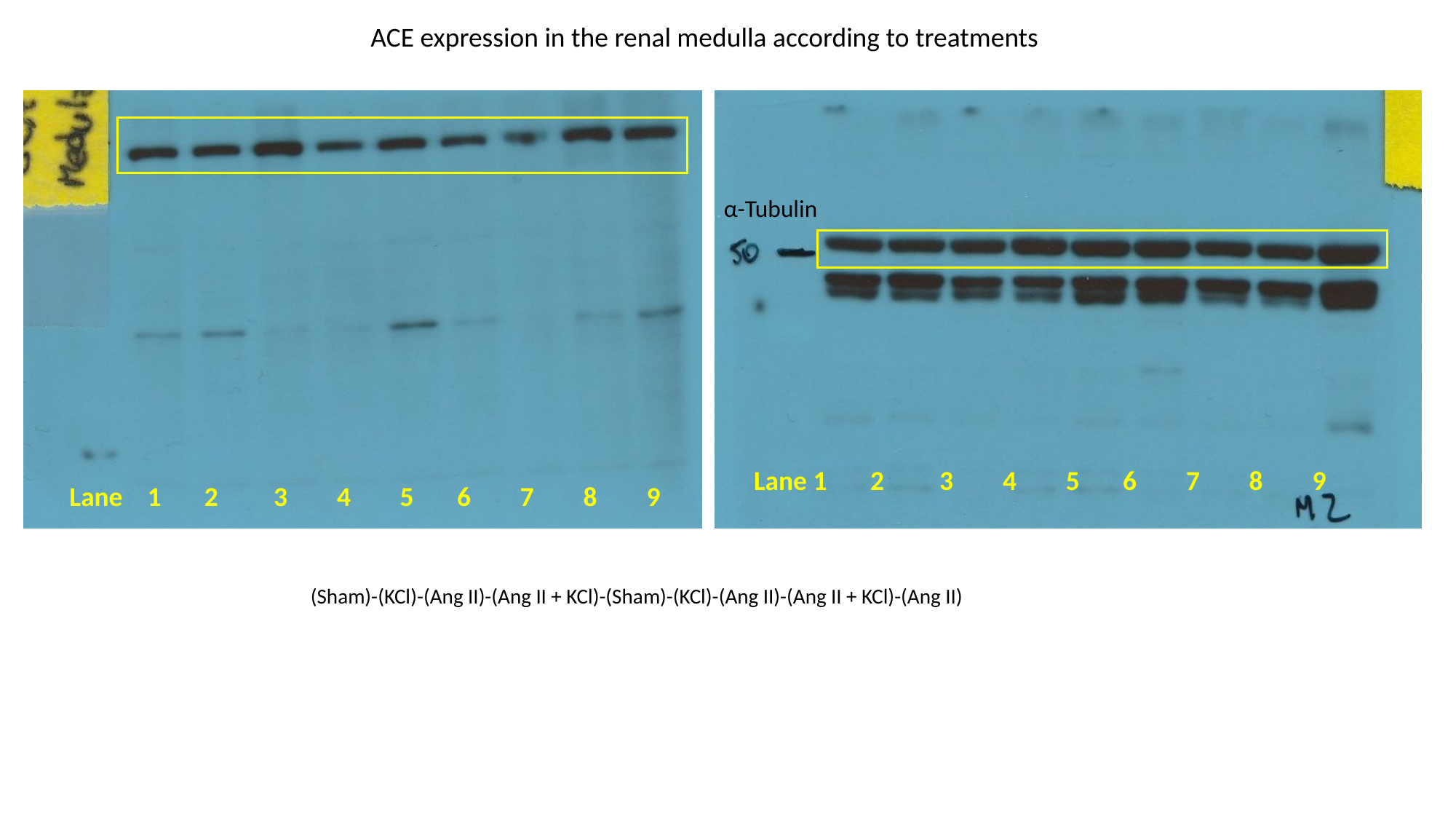

ACE expression in the renal medulla according to treatments
α-Tubulin
Lane 1 2 3 4 5 6 7 8 9
Lane 1 2 3 4 5 6 7 8 9
(Sham)-(KCl)-(Ang II)-(Ang II + KCl)-(Sham)-(KCl)-(Ang II)-(Ang II + KCl)-(Ang II)

## Slide 10
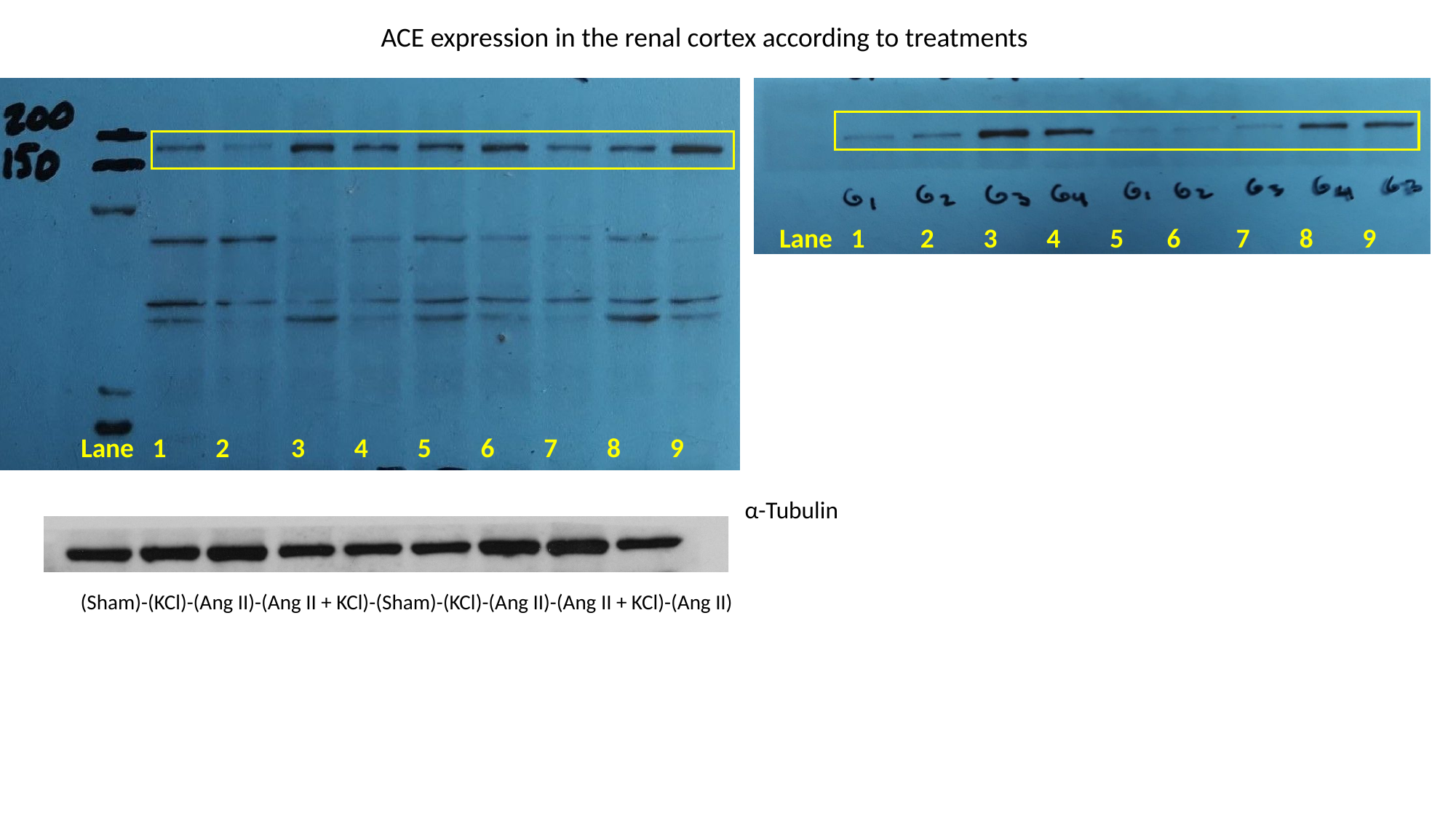

ACE expression in the renal cortex according to treatments
Lane 1 2 3 4 5 6 7 8 9
Lane 1 2 3 4 5 6 7 8 9
α-Tubulin
 (Sham)-(KCl)-(Ang II)-(Ang II + KCl)-(Sham)-(KCl)-(Ang II)-(Ang II + KCl)-(Ang II)

## Slide 11
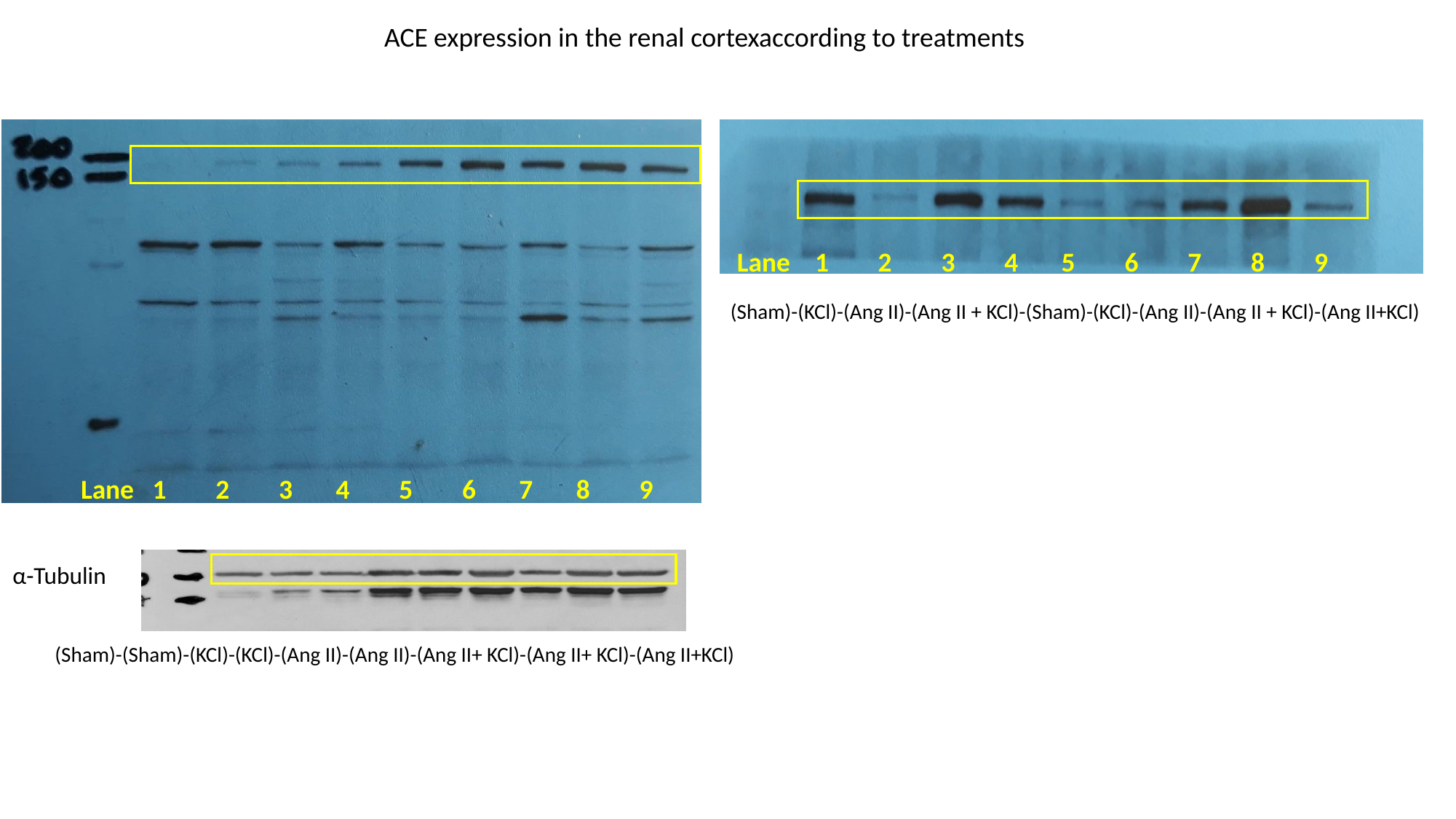

ACE expression in the renal cortexaccording to treatments
Lane 1 2 3 4 5 6 7 8 9
 (Sham)-(KCl)-(Ang II)-(Ang II + KCl)-(Sham)-(KCl)-(Ang II)-(Ang II + KCl)-(Ang II+KCl)
Lane 1 2 3 4 5 6 7 8 9
α-Tubulin
 (Sham)-(Sham)-(KCl)-(KCl)-(Ang II)-(Ang II)-(Ang II+ KCl)-(Ang II+ KCl)-(Ang II+KCl)

## Slide 12
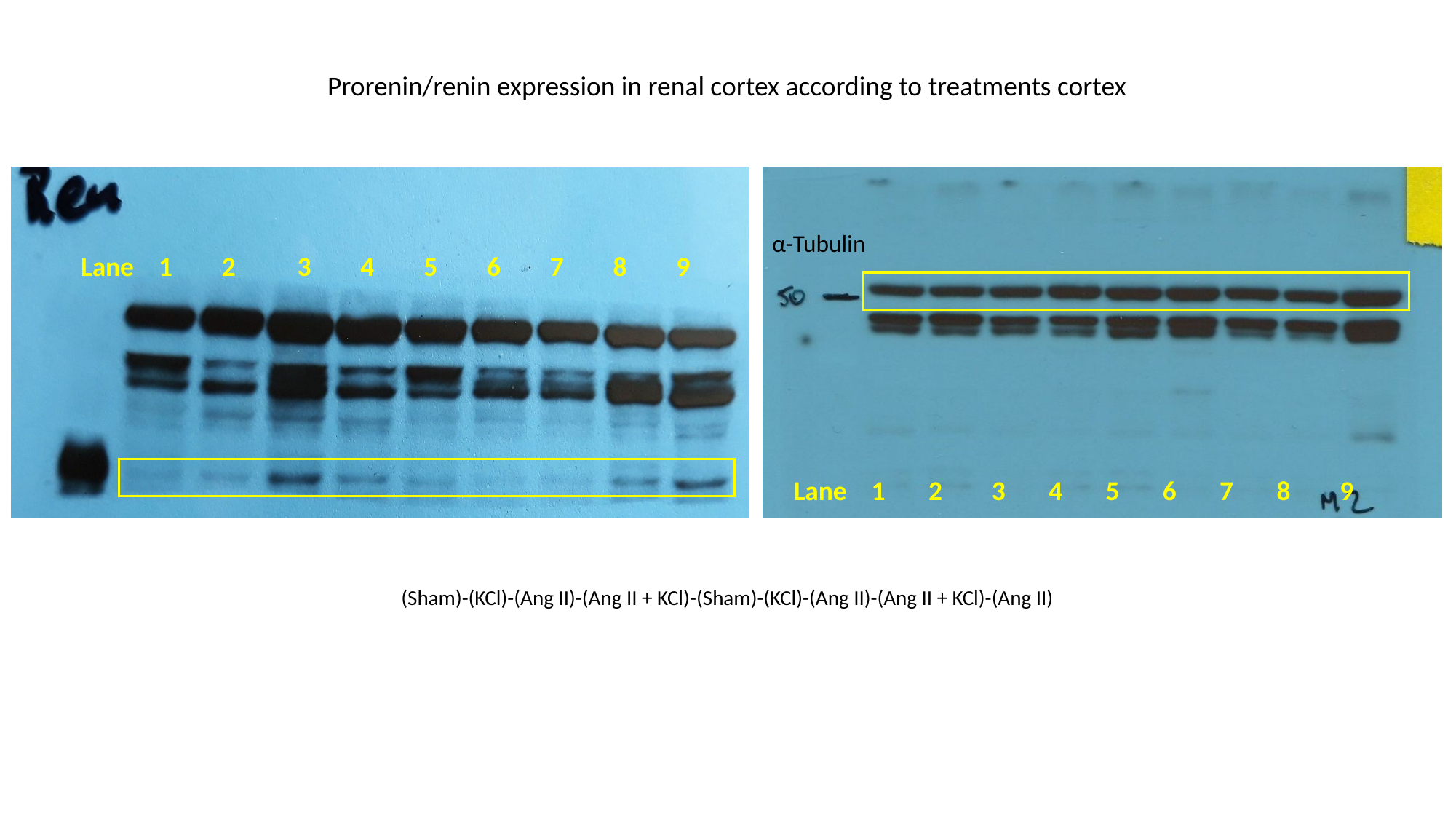

Prorenin/renin expression in renal cortex according to treatments cortex
α-Tubulin
Lane 1 2 3 4 5 6 7 8 9
Lane 1 2 3 4 5 6 7 8 9
(Sham)-(KCl)-(Ang II)-(Ang II + KCl)-(Sham)-(KCl)-(Ang II)-(Ang II + KCl)-(Ang II)

## Slide 13
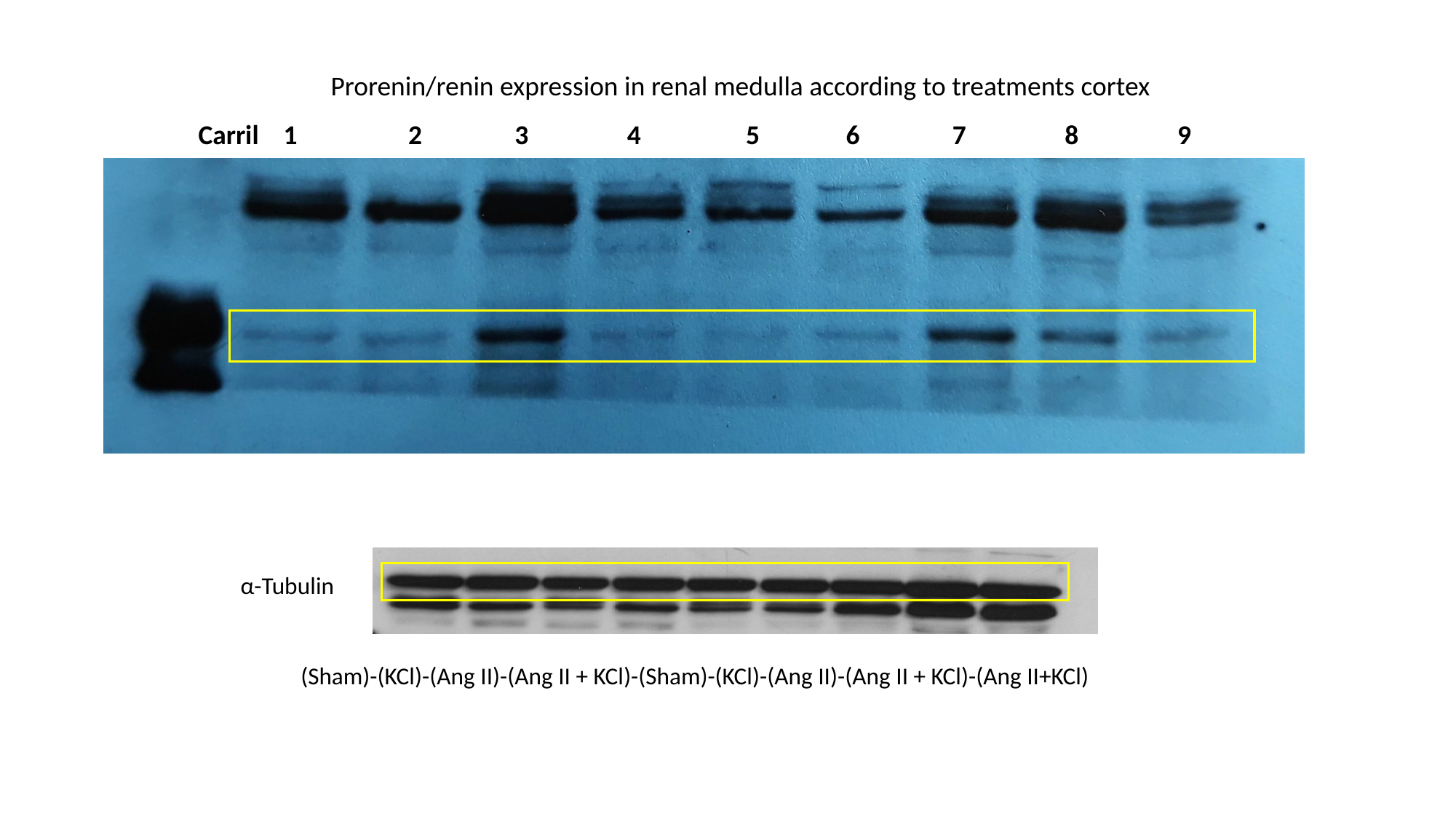

Prorenin/renin expression in renal medulla according to treatments cortex
Carril 1 2 3 4 5 6 7 8 9
α-Tubulin
(Sham)-(KCl)-(Ang II)-(Ang II + KCl)-(Sham)-(KCl)-(Ang II)-(Ang II + KCl)-(Ang II+KCl)

## Slide 14
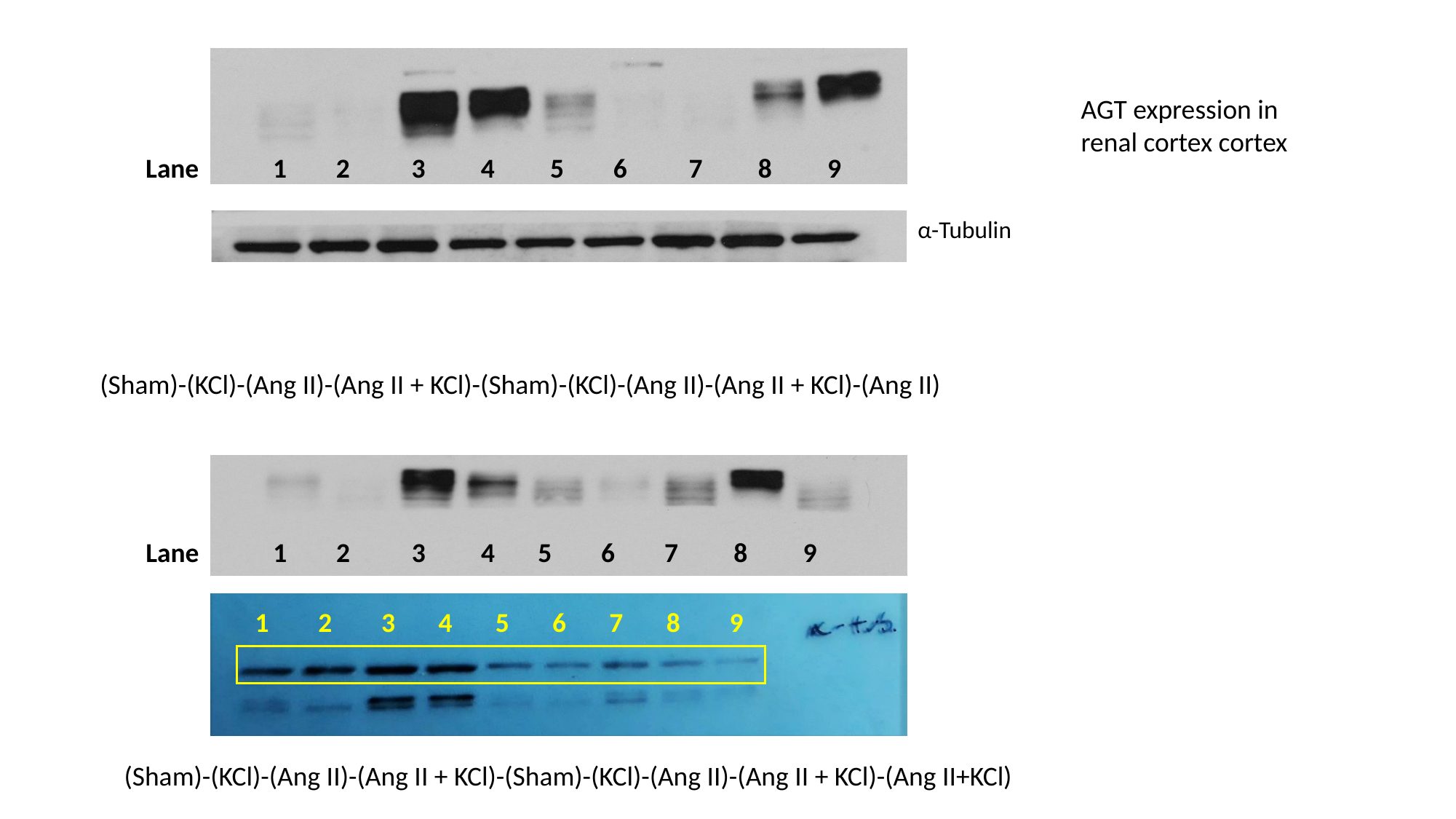

AGT expression in renal cortex cortex
Lane 1 2 3 4 5 6 7 8 9
α-Tubulin
 (Sham)-(KCl)-(Ang II)-(Ang II + KCl)-(Sham)-(KCl)-(Ang II)-(Ang II + KCl)-(Ang II)
Lane 1 2 3 4 5 6 7 8 9
 1 2 3 4 5 6 7 8 9
 (Sham)-(KCl)-(Ang II)-(Ang II + KCl)-(Sham)-(KCl)-(Ang II)-(Ang II + KCl)-(Ang II+KCl)
